# Curcumin-berberine ointment based on multifunctional carrier accelerates wound healing in a translational porcine model of tail-biting injuries: a controlled study against oxytetracycline

**DOI:** 10.3389/fphar.2026.1798208

**Published:** 2026-04-08

**Authors:** Paweł Biernat, Dominik Marciniak, Dawid Bursy, Konrad Krajewski, Radosław Balwierz

**Affiliations:** 1 Department of Drug Forms Technology, Faculty of Pharmacy, Wrocław Medical University, Wroclaw, Poland; 2 Biotts SA, Bielany Wroclawskie, Poland; 3 Faculty of Computer Science and Management, Wrocław University of Science and Technology, Wrocław, Poland; 4 Institute of Chemistry, University of Opole, Opole, Poland

**Keywords:** berberine, carrier, chronic wounds, curcumin, porcine model, translational research, wound healing

## Abstract

**Background:**

Chronic wounds represent a significant clinical burden, with limited efficacy of conventional therapies, particularly in wounds complicated by necrosis and biofilms. This study evaluated the efficacy of MTC-U1 ointment based on a patented Multifunctional Carrier containing *Curcuma longa* extract (3% w/w) and berberine hydrochloride (1.6% w/w) in a porcine model of chronic infected wounds compared to standard oxytetracycline therapy.

**Methods:**

Fourteen Polish Large White fatteners with Grade 4 tail-biting injuries were randomized into two groups (n=7): MTC-U1 ointment or oxytetracycline spray (3.84%), applied twice daily for 21 days without surgical intervention. Wound healing was assessed using a modified Wilson scoring system (0–35 points). Secondary outcomes included infrared thermography, hematological parameters (neutrophils, haptoglobin), and local tolerance.

**Results:**

The healing time (from necrotic tissue sequestration to complete scab formation) was significantly shorter in the MTC-U1 group compared to the control group (9.57 ± 2.64 vs. 13.43 ± 2.88 days; *p =* 0.023), representing a 3.86-day (29%) reduction. A statistically significant decrease in wound scores was observed in the MTC-U1 group beginning on the second day after the procedure, with a *p*-value less than 0.01. Thermographic assessment revealed significantly lower temperature at the tail base region of interest in the MTC-U1 group on Day 2 (37.2 °C ± 2.3 °C vs. 39.9 °C ± 0.7 °C; *p <* 0.05). Hematological findings confirmed the resolution of inflammation, as evidenced by the normalization of neutrophil counts in the MTC-U1 group (14.54 → 8.79 × 10^9^/L; *p* < 0.05), while they remained elevated in the control group. Serum haptoglobin levels were significantly lower in the MTC-U1 group at endpoint (0.74 ± 0.66 vs. 1.86 ± 0.87 mg/mL; *p <* 0.05). No irritation reactions were observed, as evidenced by a mean irritation index of less than 0.5.

**Conclusion:**

The MTC-U1 ointment significantly accelerated chronic wound healing compared to antibiotic therapy, demonstrating anti-inflammatory effects confirmed by thermographic and hematological assessments. These findings support further preclinical validation and clinical trials in patients with hard-to-heal wounds.

## 1 Introduction

Complex chronic wounds represent a significant dual burden, posing both clinical and economic challenges. In the United States, the prevalence of this condition is approximately 8.2 million patients annually, with associated healthcare expenditures reaching 28.1–96.8 billion dollars depending on the etiology of the wound (Sen, 2021). From a pathological perspective, these wounds are characterized by a prolonged inflammatory phase, delayed granulation, and a high susceptibility to secondary bacterial infections (Frykberg and Banks, 2015; Zhao et al., 2016). Despite advances in tissue bioengineering and growth factor therapies, available topical formulations remain suboptimal for wounds complicated by necrosis and bacterial biofilms (Dhivya et al., 2015). The escalating antimicrobial resistance (AMR) crisis further restricts therapeutic options, highlighting the need for alternative strategies independent of conventional antibiotics (Sen, 2021).

The domestic pig (*Sus scrofa domesticus*) is a well-established translational model in wound healing research owing to its high structural and functional homology with human skin, including comparable epidermal thickness (30–140 µm), collagen type I/III ratio, hair follicle density, and microvascular architecture (Sullivan et al., 2001; Seaton et al., 2015; Elloso et al., 2025). Collectively, these characteristics determine the penetration of active agents, the kinetics of the inflammatory process, and the dynamics of healing. Naturally occurring tail-biting injuries in intensive swine production settings serve as a clinically relevant model of chronic wounds. These lesions exhibit a combination of characteristics associated with lacerations and contusions, accompanied by inflammation, tissue necrosis, and the potential for secondary bacterial infections that can progress to systemic sepsis (Taylor et al., 2010; D’Eath et al., 2014). Porcine wounds have been identified as a well-established research model due to their propensity to form bacterial biofilms *in vivo*, a process analogous to that observed in human wounds (Davis et al., 2008). It is estimated that biofilms are present in 78% to over 90% of human chronic wounds (Davis et al., 2008; Malone et al., 2017). Bacteria within these structures exhibit significantly higher antibiotic tolerance compared to planktonic forms, as the biofilm architecture impedes antimicrobial penetration and shields pathogens from the host immune system (Li et al., 2023). This phenomenon is a primary driver of wound chronicity and frequently leads to the failure of conventional antibiotic therapy (Li et al., 2023; Davis et al., 2025). Consequently, porcine tail wounds offer a realistic, non-sterile testing environment that closely mirrors the pathophysiology of pressure ulcers and diabetic foot ulcers (Sullivan et al., 2001; Seaton et al., 2015; Davis et al., 2025; Elloso et al., 2025).

*Curcuma longa* (Turmeric) and *Coptis chinensis* (Chinese Goldthread) are plants with well-documented anti-inflammatory, antibacterial, and wound-healing properties (Akbik et al., 2014; Neag et al., 2018; Kandaswamy et al., 2024). Curcumin, the primary curcuminoid found in the turmeric rhizome, has been shown to exert inhibitory effects on cyclooxygenase-2 (COX-2), 5-lipoxygenase (5-LOX), and the nuclear factor kappa B (NF-κB) transcription factor. This results in a reduction of the synthesis of pro-inflammatory prostaglandins and leukotrienes (Prasad et al., 2014). In contrast, berberine facilitates the polarization of macrophages from the pro-inflammatory M1 phenotype to the pro-reparative M2 phenotype, a shift that is essential for the progression from the inflammatory to the proliferative phase of wound healing (Li et al., 2023). In addition, berberine-loaded liposomes disrupt bacterial quorum sensing and compromise the integrity of the biofilm extracellular polymeric substance (EPS) matrix, thereby enhancing bacterial eradication efficacy compared to conventional antibiotics such as ciprofloxacin or ceftazidime (Li et al., 2023). Isoquinoline alkaloids present in *Coptis chinensis*, particularly berberine, are characterized by a broad spectrum of antibacterial activity encompassing both Gram-positive (*Staphylococcus* spp., *Streptococcus* spp.) and Gram-negative bacteria (*Escherichia coli*, *Pseudomonas aeruginosa*). In addition to their antibacterial properties, these alkaloids have been observed to exert anti-inflammatory effects via the inhibition of the NF-κB/MAPK signaling pathway (Neag et al., 2018; Zhou et al., 2021). These findings align with recent studies emphasizing the renaissance of ethnopharmacological approaches in wound management and highlighting the efficacy of multi-herbal formulations in overcoming antibiotic resistance (Paul-Traversaz et al., 2023). Specifically, the incorporation of alkaloid-rich extracts targets the biofilm architecture that is often responsible for the chronicity of infected wounds. For instance, berberine-loaded hydrogels have demonstrated significant potential in eradicating mature biofilms of *S. aureus* and *P. aeruginosa*, thereby restoring the healing trajectory in infected cutaneous defects (Li et al., 2023).

Ensuring adequate bioavailability of active agents within target tissues remains a critical challenge in the field of topical wound management. To address this limitation, a proprietary *Multifunctional Transdermal Carrier* (MTC) was developed, utilizing dimethyl sulfoxide (DMSO) and urea as synergistic percutaneous penetration enhancers (Biotts SA, 2024; Biotts SA, 2025). This system has previously been employed for the effective delivery of anesthetics (Biernat et al., 2025). DMSO disrupts intercellular lipid packing within the stratum corneum via hydrogen bonding with ceramides and cholesterol (Williams and Barry, 2004; Schafer et al., 2023), whereas urea promotes keratin hydration and the delamination of corneocyte layers (Celleno, 2018). Preliminary *ex vivo* studies on intact porcine skin explants demonstrated that the optimized MTC-U(Na) formulation (pH 7.4) yielded a 72% increase in stratum corneum thickness and a 3.2-fold enhancement in membrane permeability over 12 h, without evidence of cytotoxicity (see Section 3.1 for complete data). These structural modifications facilitate the dermal penetration of curcumin (MW 368 Da, log P 3.2) and berberine (MW 336 Da, log P 3.6), thereby circumventing the pharmacokinetic hurdles that have historically impeded the clinical translation of these agents. Furthermore, the formulation vehicle incorporates Chaulmoogra oil (*Hydnocarpus wightianus*), a rich source of cyclic unsaturated fatty acids (chaulmoogric acid, hydnocarpic acid) exhibiting potential antimicrobial properties (Schäfer et al., 2025; Schäfer et al., 2025) and zinc sulfate, a cofactor for extracellular matrix metalloproteinases involved in tissue remodeling (Lansdown et al., 2007). In conclusion, the occlusive nature of the lipid vehicle (porcine lard, goose fat, and white petrolatum) maintains a moist wound microenvironment, a prerequisite for optimal keratinocyte migration and fibroblast proliferation. This aligns with Winter’s concept of moist wound healing (Winter, 1962).

Oxytetracycline, a member of the broad-spectrum tetracycline antibiotic class, is frequently utilized in the management of wounds in veterinary contexts. The use of oxytetracycline in chronic infected wounds is limited by escalating antibiotic resistance, notably among *Staphylococcus aureus* strains, and the potential for suboptimal concentrations within the wound bed. Such suboptimal concentrations may, in contrast, serve as a catalyst for the development of resistance (Kim and Kim, 2024). These constraints, in conjunction with the imperative for immune response modulation, underscore the need for alternative therapeutic strategies such as curcumin and berberine, which not only disrupt biofilm structure but also promote macrophage polarization toward the pro-reparative M2 phenotype (Prasad et al., 2014; Li et al., 2023).

Therefore, the aim of this controlled preclinical study was to assess whether the MTC-mediated enhancement of curcumin and berberine bioavailability leads to accelerated wound healing in naturally occurring tail-biting injuries in pigs, a clinically relevant model of chronic polymicrobial wounds. The following hypothesis was formulated: the MTC-U1 ointment, containing extract of *Curcuma longa* (3% w/w) and berberine hydrochloride (1.6% w/w) within the MTC vehicle, would: (1) attenuate local inflammation, as quantified by infrared thermography and systemic acute-phase protein markers (haptoglobin, neutrophil count); (2) accelerate wound closure time by at least 20% compared to standard topical oxytetracycline therapy; and (3) promote moist healing, as evidenced by clinical observation of granulation tissue and reduced crust formation. In light of the rising concerns regarding antimicrobial resistance in both veterinary and human medicine, a secondary objective was established to assess whether MTC-U1 could serve as an antibiotic-sparing alternative for the management of infected chronic wounds. The primary endpoint was defined as the time interval from the onset of necrotic tissue sequestration to the complete formation of scabs. Secondary endpoints included thermographic assessment of local inflammation, systemic inflammatory markers (haptoglobin, neutrophil count), and local tolerance evaluation. Consequently, the findings of this study hold translational significance for the management of hard-to-heal wounds in both veterinary and human populations, particularly in the context of venous ulcers, diabetic foot syndrome, and pressure ulcers, where conventional topical antibiotic therapy faces increasing limitations due to AMR.

## 2 Materials and methods

### 2.1 Ethical statement

The study was conducted in strict compliance with Directive 2010/63/EU of the European Parliament and of the Council on the protection of animals used for scientific purposes and followed the ARRIVE 2.0 guidelines for reporting animal research. The study protocol was approved by the Local Ethics Committee for Animal Experiments in Poznań (Resolution No. 18/2020 of June 26, 2020).

### 2.2 Investigational product

The MTC-U1 formulation was developed and manufactured by Biotts S.A. (Wrocław, Poland) utilizing patented technology (Polish patent PL245223B1 (Biotts SA, 2024; Biotts SA, 2025). The ointment is formulated with two primary active ingredients: 3.0% (w/w) *Curcuma longa* extract (water-soluble form; curcuminoid content ≥20%, batch No. 20190628; ASTI Plus s.c., Poland), and 1.6% (w/w) berberine hydrochloride (C_20_H_18_ClNO_4_·2H_2_O; assay 98.0% on anhydrous basis; batch No. C025B190702; Sichuan Xieli Pharmaceutical Co., Ltd., China; compliant with Japanese Pharmacopoeia 14th edition). The quality specifications of the active ingredients were verified by Certificates of Analysis (CoA) provided by the manufacturers. These agents are delivered via a specialized patented carrier system (MTC) composed of dimethyl sulfoxide (DMSO), which acts as an aprotic solvent facilitating percutaneous penetration; urea, serving as a keratolytic and moisturizing agent; white beeswax (*Cera alba*) acting as a natural emulsifier; and Chaulmoogra oil (*Hydnocarpus wightianus*), a source of cyclic unsaturated fatty acids such as chaulmoogric and hydnocarpic acids. The formulation vehicle consists of a lipid base comprising porcine lard and goose fat, supplemented with Eucerin as an emulsifying agent and zinc sulfate, included for its antiseptic properties and ability to promote epithelialization. The precise weight ratios of the active substances to the carrier, as well as the detailed quantitative proportions of the excipients, constitute proprietary information protected by the aforementioned patents.

#### 2.2.1 *Ex vivo* characterization of the carrier system

A complementary *ex vivo* study was conducted to evaluate the structural effects of the carrier system on the skin barrier prior to the incorporation of active ingredients. Intact porcine skin explants were utilized for this purpose. This investigation was essential to differentiate carrier-mediated effects from the pharmacological activities of curcumin and berberine. Fresh full-thickness porcine skin biopsies (dorsal region, n=18 samples from 3 animals) were obtained from an accredited abattoir and immediately transported on ice (4 °C) in sterile phosphate-buffered saline (PBS). The skin samples (2 cm × 2 cm) were mounted in Franz diffusion cells (diffusion area 2.5 cm^2^) with the *stratum corneum* facing the donor compartment within 4 h of tissue harvest. Three carrier variants were evaluated: MTC-U (standard), MTC-U(Na) (pH-optimized with sodium hydroxide), and MTC-U(A) (acidified with citric acid). The carriers were applied topically (200 μL/cm^2^), and the samples were subsequently incubated at 37 °C for 12 h. At designated time points of 1, 3, 6, and 12 h, the samples were meticulously washed with phosphate-buffered saline (PBS), then fixed in 4% paraformaldehyde, and subsequently embedded in OCT compound. Cryosections (10 μm) were subjected to double staining with 4′,6-diamidino-2-phenylindole (DAPI; 1 μg/mL; for nuclear staining) and propidium iodide (PI; 5 μg/mL; for membrane-compromised cells). Imaging was performed using an Olympus BX53 fluorescence microscope equipped with a 20 × objective (NA 0.75). The thickness of the *stratum corneum* was measured using ImageJ software (n = 10 measurements per sample). PI fluorescence intensity was quantified as an index of membrane permeability. Furthermore, the extent of stratum corneum delamination was evaluated using a semi-quantitative scale, ranging from 0 (absence of delamination) to 3 (severe delamination). As the skin samples were by-products of commercial meat production, no additional ethical approval was required in accordance with EU Directive 2010/63/EU, as the animals were not sacrificed specifically for experimental purposes.

### 2.3 Control formulation

Engemycin Spray 3.84% (MSD Animal Health) was employed as the positive control. This formulation contains oxytetracycline, a broad-spectrum tetracycline antibiotic that serves as the current standard for wound management in livestock. The decision to omit a placebo arm was made in the interest of ethical considerations, particularly with regard to the management of chronic infected wounds that remained untreated. This design choice is recognized as a limitation of the study.

### 2.4 Animals

The present study was conducted on a group of 14 Polish Large White (PLW) fatteners of both sexes, with an average age of approximately 10–12 weeks at the time of enrolment. Animals were selected 24–48 h following the onset of acute tail-biting episodes, as confirmed by farm records. The animals were sourced from a parent farm with a documented history of tail biting induced by high stocking density, which manifested 3 weeks prior to the study initiation. The following criteria were used to determine the eligibility of subjects for participation in the study: Tail lesions were classified as Grade 4 on a 5-point tail lesion scoring scale (Table 1). The presence of tail tip necrosis or autotomy occurring within 24 h prior to the experiment has been documented. No prior surgical intervention (i.e., tail docking) had been performed at the farm of origin. The subject’s general clinical status was satisfactory, with the exception of a lesion on the tail.

**TABLE 1:** Porcine tail lesion classification scale (0–4).

Grade	Description of lesions
0	No signs of tail biting
1	Superficial skin abrasions; no visible open wounds, edema, or necrosis
2	Fresh wound: Evidence of biting, puncture wounds on the tip, epidermal damage, erythema; no edema
3	Evidence of chewing or puncture wounds, erythema, edema; no tissue necrosis
4	Total tail loss or tip necrosis, open wound, erythema, edema, tissue necrosis, purulent exudate, eschar formation

The following data were collected on the first day of the study to establish a baseline for future analysis. The control group (n = 7) is defined as follows: The mean body weight (BW) was 25.32 ± 2.16 kg, and the male-to-female ratio was 3:4. The MTC-U1 Group (n = 7): The mean body weight (BW) was 31.46 ± 6.26 kg, and the male-to-female ratio was 4:3. A *post hoc* power analysis for the observed body weight differences yielded a power (1-β) of 0.62 (two-sided α = 0.05). Despite this value falling below the conventional 0.80 threshold, the large effect size (Cohen’s *d =* 1.31) enabled the detection of statistical significance despite the limited sample size (n = 7). The mean length of the viable tail segment (measured from the base to the vital/necrotic tissue demarcation line) was 5.2 ± 3.6 cm, compared to an estimated total tail length of approximately 18 cm, which is typical for this age and weight category.

### 2.5 Housing and husbandry conditions

The animals were housed in pens with a floor area of 6.0 m^2^ featuring fully slatted flooring, in strict compliance with Council Directive 2008/120/EC and the Regulation of the Polish Minister of Agriculture and Rural Development of 15 February 2010. The environmental parameters were subject to continuous monitoring to ensure stability, and the results indicated a mean air temperature of 21.4 °C ± 1.2 °C and relative humidity of 52.5% ± 3.0%. Gas concentrations were meticulously regulated, with maximal levels of CO_2_ and NH_3_ reaching 1596 ppm and 12.8 ppm, respectively, while H_2_S concentrations remained below the limit of detection. With regard to pen furnishings, each unit was equipped with a single feeder, two bowl drinkers positioned at different heights, and four environmental enrichment devices, in accordance with Commission Recommendation (EU) 2016/336. Throughout the experiment, the animals were provided with a complete standard diet *ad libitum* and granted unrestricted access to drinking water that met quality standards for human consumption.

### 2.6 Experimental design

**FIGURE 1:**
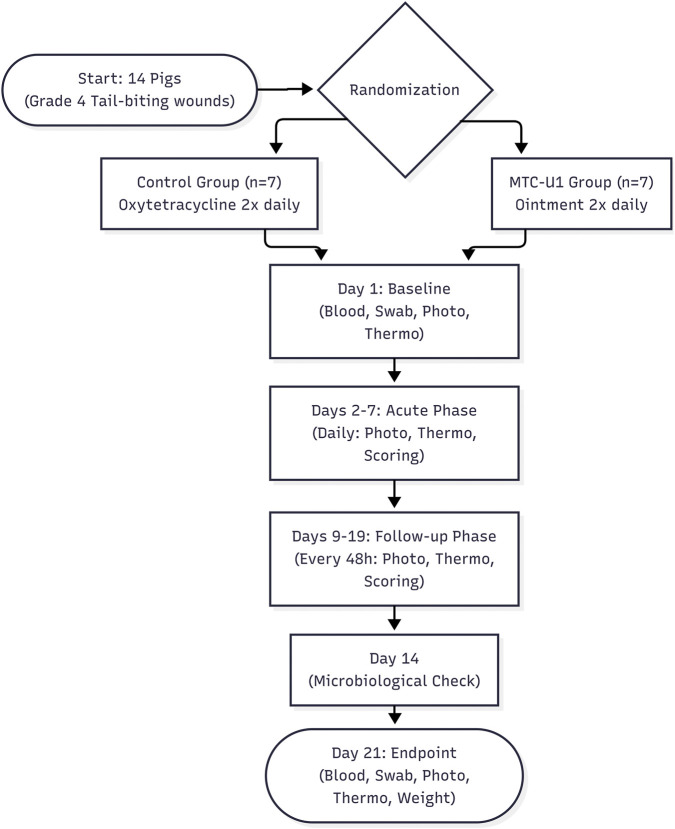
Schematic representation of the experimental design. The timeline illustrates the randomization of 14 pigs into two treatment arms: MTC-U1 (investigational ointment) and Control (oxytetracycline spray). The protocol comprised an Acute Phase (Days 2–7) with daily assessments to monitor rapid inflammatory dynamics, followed by a Follow-up Phase (Days 9–19) with assessments every 48 h. Samples were obtained from the subjects at three distinct time points: at the baseline, on Day 14, and on Day 21, which corresponded to the endpoint of the study. These samples included blood and swabs, and their analysis was used to assess the systemic safety of the treatment and the local antimicrobial efficacy.

The animals were divided into two experimental groups through a block randomization procedure stratified by the baseline severity of tail lesions to ensure comparability between cohorts. The randomisation process was performed by D.M. using a computer-generated sequence. The control group (n = 7) received topical oxytetracycline therapy (Engemycin Spray 3.84%), whereas the MTC-U1 group (n = 7) was treated with the investigational MTC-U1 ointment. The MTC-U1 ointment was administered at approximately 0.5 g per application (sufficient to cover the wound bed with a 2-mm layer), while Engemycin spray was applied according to manufacturer’s instructions (2-s spray from 15 cm distance, approximately 0.3 mL per application). Application amounts were not adjusted during the study period. The therapeutic regimen was administered twice daily (morning and evening) over a period of 21 consecutive days. Prior to each administration of the pharmaceutical agents, the wound area was meticulously cleansed with a sterile 0.9% saline solution. No surgical interventions were performed during the study. Invasive procedures, such as surgical debridement, excision of necrotic tissue, or mechanical removal of eschars, were strictly prohibited to isolate the pharmacological effect of the tested formulations. The study protocol established two distinct monitoring periods in order to evaluate the temporal progression of healing. The first period was designated as the “Acute Phase,” which occurred from days 2–7. During this phase, daily assessments were performed. The second period was designated as the “Follow-up Phase,” which occurred from days 9–19. During this phase, evaluations were performed every 48 h. Furthermore, biological samples (e.g., blood and swabs) were collected at predetermined intervals (i.e., baseline, Day 14, and endpoint) to evaluate systemic safety and local antimicrobial efficacy, as illustrated schematically in Figure 1.

### 2.7 Clinical assessment of wound healing

Wound healing progression was assessed daily during the initial acute phase (Days 1–7) and subsequently at 48-h intervals until the study’s conclusion on Day 21. Quantitative assessment was performed using the semi-quantitative scoring system established by Wilson et al. (Wilson et al., 1986), as detailed in Table 2.

**TABLE 2:** Clinical wound scoring parameters (adapted from (Wilson et al., 1986))

Parameter	Scoring range
Wound size (relative to the tail segment diameter)	0–5
Presence of serous exudate	0–5
Erythema (redness)	0–5
Purulent exudate	0–10
Necrotic changes	0–10
Total maximum score	35

To ensure terminological consistency throughout this manuscript, the following wound surface descriptors are defined. An eschar refers to a layer of devitalized necrotic tissue that forms over the wound bed as a consequence of tissue death; in the present model, eschar was the predominant wound surface feature at study entry (Grade 4 lesions). Upon demarcation and detachment of the eschar (necrotic tissue sequestration), the exposed wound bed undergoes progressive granulation. A scab (used interchangeably with crust) denotes the dried protective covering composed of desiccated wound exudate, fibrin, and cellular debris that forms over the granulating wound bed during the reparative phase. Critically, eschar and scab represent fundamentally distinct biological processes: the former is a pathological remnant of tissue necrosis, whereas the latter is a physiological component of wound repair.

Additionally, the temporal sequence of critical healing milestones was meticulously monitored and documented for each subject. Firstly, the necrotic tissue sequestration process, also known as the demarcation phase, must be completed. The second phase is the emergence of granulation tissue, also known as the granulation phase. The third component of the healing process is the formation of a protective crust (scab), which is a thick, yellowish-white dried exudate covering that forms over the entire wound bed. The last component is crust detachment and completion of re-epithelialization. Standardized photographic documentation was obtained on Days 1, 3, 5, 7, 9, 11, 13, 15, 17, 19, and 21 to visualize the healing trajectory. Due to the distinct physical appearance of the treatments (ointment vs. spray), complete blinding of the personnel administering the drugs was not feasible. However, to minimize detection bias, the photographic documentation was anonymized and scored retrospectively by two independent investigators who were unaware of the treatment allocation at the time of assessment. Photographs were presented to assessors in a randomized order to prevent temporal bias.

### 2.8 Infrared thermography assessment

Surface body temperature profiles were quantified using a FLIR ONE PRO thermal imager (FLIR Systems, United States). To ensure data reproducibility, image acquisition was conducted at a standardized distance of 1.0 m under controlled ambient environmental conditions. Ambient barn temperature (21.4 °C ± 1.2 °C) and relative humidity (52.5% ± 3.0%) were continuously monitored throughout the study period to ensure that external environmental factors did not confound thermographic readings. Both treatment groups were housed in the same facility under identical environmental conditions. Thermographic monitoring was conducted in accordance with the clinical assessment schedule, occurring on Days 1, 2, 3, 4, 5, 6, 7, 9, 11, 13, 15, 17, 19, and 21. Subsequent to the acquisition, a detailed analysis was conducted utilizing FLIR Tools software. For each thermogram, two distinct Regions of Interest (ROIs) were defined to evaluate specific pathological and physiological processes: (1) Proximal Tail Base ROI: To assess peri-caudal inflammation and tissue hyperemia. (2) Viable Distal Tip ROI: To evaluate perfusion levels within the regenerating tissue. For each ROI, the mean, maximum, and minimum temperatures were extracted. The data obtained from age-matched, clinically healthy pigs with intact tails served as the baseline reference values.

### 2.9 Hematological and microbiological assessment

To evaluate systemic inflammatory responses and general physiological status, comprehensive laboratory profiling was conducted at the beginning (Day 1) and conclusion (Day 21) of the experiment. Peripheral blood samples were analyzed using an automated ABC Vet analyzer (Horiba ABX, France). The hematological panel encompassed a complete erythrocyte profile (RBC, Hb, Ht, MCV, MCH, MCHC) as well as a total and differential leukocyte count (WBC), quantifying neutrophils, lymphocytes, monocytes, eosinophils, and basophils. Concurrently, the acute-phase response was monitored by determining levels of haptoglobin (via immunoturbidimetry), fibrinogen, total protein, albumin, and globulin fractions. All values obtained were interpreted against standard reference intervals for swine (Sipos et al., 2011; Bhattarai et al., 2019) specifically: WBC 11.0–22.0 × 10^9^/L, neutrophils 3.0–10.0 × 10^9^/L (28%–47%), lymphocytes 4.2–13.6 × 10^9^/L (39%–62%), monocytes 0.2–2.2 × 10^9^/L (2%–10%), and haptoglobin levels reaching up to approximately 1.0 mg/mL.

In parallel, microbiological surveillance was conducted to evaluate the local effectiveness of antimicrobials and the microbial bioburden. Wound swabs were collected on Days 1, 14, and 21 and subsequently inoculated onto Blood Agar, MacConkey Agar, and Chapman Agar (bioMérieux, France). Cultures were subjected to incubation under both aerobic and microaerophilic conditions to ensure comprehensive pathogen detection. Following the incubation period, the bacterial isolates were identified through the implementation of standard biochemical methods. The determination of their antimicrobial susceptibility profiles was conducted via the disk diffusion technique.

### 2.10 Safety and local tolerance assessment

The local dermal tolerance of the MTC-U1 formulation was evaluated through daily clinical observations of the application site (perineal and caudal regions), conducted 30 min post-administration. A meticulous inspection of the skin was conducted to ascertain the presence of adverse reactions, with particular attention devoted to the observation of erythema (redness), dermal lesions, oedema, and indications of pruritus (itching/discomfort). The quantification of the irritation potential was conducted by calculating the Mean Irritation Index (x_*av*_), in accordance with the ISO 10993-10 standard. According to the established classification system, a formulation yielding an index value of <0.5 is designated as non-irritating.

### 2.11 Zootechnical performance indicators

In order to provide additional insight into the general wellbeing of the animals and the overall health status of the system, the key production parameters were closely monitored. The body weight (BW) of the animals was documented by means of individual weigh-ins at the study’s baseline (Day 1) and endpoint (Day 21). Subsequently, the Feed Conversion Ratio (FCR) was calculated for the entire experimental period, defined as the ratio of total feed intake [kg] to total body weight gain [kg].

### 2.12 Statistical analysis

Each animal presented with a single Grade 4 tail lesion at the distal tail segment, which constituted the unit of analysis. Consequently, the statistical unit was the individual animal (n = 7 per group), and no correction for intra-animal correlation was necessary. Statistical analyses were conducted using TIBCO Statistica™ version 13.3 (TIBCO Software Inc., Palo Alto, CA, United States). The normality of distribution for continuous variables was verified using the Shapiro-Wilk test, while the homogeneity of variances was assessed via Levene’s test. For the purpose of inter-group comparisons of continuous variables exhibiting a normal distribution, the Student’s t-test for independent samples was applied, assuming equal variances. In instances of variance heterogeneity (F-ratio ≥4), the Welch’s t-test with Satterthwaite’s degree of freedom correction was utilized. To evaluate the temporal dynamics of hematological parameters within the same subjects (Day 1 vs. Day 21), the paired Student’s t-test was employed. The parametric data are expressed as mean ± standard deviation (SD). Non-parametric tests were applied to variables violating assumptions of normality or in cases of small sample sizes. Multiple comparisons between groups across different time points (specifically for the *ex vivo* carrier study) were performed using the Kruskal–Wallis test. Following the identification of substantial discrepancies, a *post hoc* analysis was conducted employing Dunn’s test with Bonferroni correction. The Mann-Whitney U test was employed to execute inter-group comparisons for two independent samples. Non-parametric data are presented as the median accompanied by quartiles (25th and 75th percentiles) or range (minimum–maximum). The effect of the treatment on final body weight was analyzed using Analysis of Covariance (ANCOVA), with baseline body weight included as a covariate. Prior to this analysis, the assumption of homogeneity of regression slopes was verified by testing the interaction between the treatment group and the covariate (*p >* 0.05). The effect size for the primary endpoint (healing time) was quantified as the standardized mean difference (Cohen’s d), with interpretation thresholds defined as: d < 0.5 (small effect), 0.5 ≤ d < 0.8 (medium effect), and d ≥ 0.8 (large effect). Furthermore, a *post hoc* power analysis was conducted to determine the statistical power of the study given the sample size (n = 7) and the observed effect size. The confidence intervals (95% CI) for mean differences were calculated using the classical method based on the t-distribution. The statistical significance was established at α = 0.05 (two-tailed). It should be noted that all statistical tests were two-sided unless otherwise specified.

## 3 Results

### 3.1 Carrier-mediated modulation of skin barrier structure (*ex vivo* study)

**FIGURE 2:**
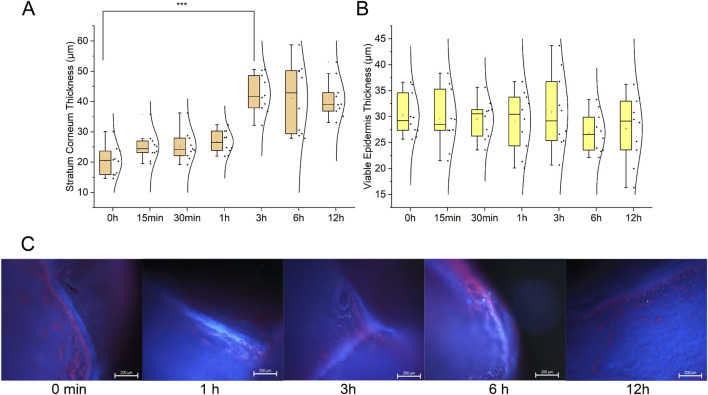
Time-dependent alterations in epidermal structure following MTC-U(Na) carrier application. **(A,B)** Thickness of the stratum corneum **(A)** and viable epidermis **(B)** in intact porcine skin samples over a 12-h observation period. The MTC-U(Na) carrier induced significant thickening of the stratum corneum (max. at 3 h, ***p < 0.001 vs. 0 h) while maintaining stable thickness of the viable epidermis. Data are presented as median, 25%–75% quartiles (box), and min-max (whiskers); n = 18 samples. **(C)** Representative fluorescence microscopy images (DAPI–blue, nuclei; PI–red, membrane permeability) demonstrating progressive hydration of the stratum corneum and increased membrane permeability in the viable epidermis without cytotoxicity. These structural changes indicate selective modulation of the skin barrier by the carrier, providing a mechanism for the enhanced curcumin/berberine penetration observed *in vivo*. Complete data for all carrier variants are presented in Supplementary Materials (Supplementary Figure S1; Supplementary Table S1; Supplementary Figures S2–S7).

To elucidate the carrier-mediated alterations in the skin barrier that potentially facilitate the penetration of curcumin and berberine, we evaluated the impact of the MTC-U carrier system on intact porcine epidermis over a 12-h period using fluorescence microscopy (Figure 2).

The application of the MTC-U(Na) carrier induced progressive thickening of the stratum corneum (SC) within the first 3 h. The median SC thickness increased from a baseline of 18 μm–45 μm at the 3-h mark (***p < 0.001 vs. 0 h; Kruskal–Wallis test with Dunn’s *post hoc* correction; n = 18 samples). This thickening was consistent with water uptake and SC swelling, a classic effect of occlusion. After 3 hours, a gradual decrease in thickness was observed. By 12 h, the presence of moderate delamination of the outer corneocyte layers was evident in microscopic images (Figure 2A). In contrast, the thickness of the viable epidermis remained stable throughout the 12-h duration (median 28–30 μm, Figure 2B), indicating the preservation of cellular integrity in the stratum basale and stratum spinosum despite significant alterations in the horny layer. The use of propidium iodide (PI), a marker for membrane integrity, revealed a time-dependent increase in PI penetration into the stratum spinosum (Figure 2C). PI fluorescence intensity increased progressively starting from hour 3, peaking at hour 12, which was manifested as distinct pink-red staining of the viable epidermal layers. Notably, no nuclear morphological distortions were detected in PI-positive regions (DAPI staining remained normal/intact), suggesting that the observed increase in PI permeability reflects enhanced cell membrane permeability rather than toxicity. Three carrier variants were assessed in the comprehensive *ex vivo* study (comparative data provided in the Supplementary Materials, Supplementary Figures S1–S3; Supplementary Tables S1,S2). Among these, the MTC-U(Na) variant (pH 7.4) exhibited the most potent effect on SC modulation and was consequently selected as the vehicle for the MTC-U1 formulation utilized in the *in vivo* wound healing study.

### 3.2 Baseline wound characteristics (Day 1)

All enrolled subjects (n = 14) presented with Grade 4 tail lesions upon study entry. The pathology was characterized by distal tail necrosis or traumatic auto-amputation occurring within the 24-h window preceding study initiation. Clinically, the wounds exhibited hemorrhagic traces, gross environmental contamination, and overt signs of bacterial infection. A subsequent statistical analysis of the baseline cumulative wound scores (Day 1) revealed no significant differences between the cohorts, ensuring homogeneity at the start of the treatment (Control: 20.86 ± 3.58 points vs. MTC-U1: 20.29 ± 2.56 points; *p =* 0.88). Regarding specific parameters, the wound size score (necrotic diameter relative to tail segment) reached the scale maximum of 5.0 in both treatment arms. Peri-wound erythema was quantified as 3.00 in the Control group and 2.86 in the MTC-U1 group.

### 3.3 Clinical wound healing progression

Both treatment arms demonstrated progressive wound resolution throughout the 21-day observation period (Table 3). Notably, the Control group exhibited a transient clinical exacerbation on Day 2, evidenced by an increase in the mean cumulative score from 20.86 to 22.00. This phenomenon likely reflects an acute inflammatory surge preceding the physiological sequestration of necrotic tissue. In contrast, the MTC-U1 cohort displayed a steady downward trajectory in wound scores, effectively mitigating this initial inflammatory spike. The acute phase (Days 1–7) was clinically characterized by the demarcation and sequestration of necrotic lesions. A statistically significant divergence between the groups was established by Day 7, where the MTC-U1 group achieved a markedly lower wound score (6.57 ± 4.12) compared to the Control group (10.86 ± 1.68; *p <* 0.05). This advantage persisted into Day 9 (*p =* 0.02), indicating a faster onset of healing in the investigational group.

**TABLE 3:** Longitudinal evolution of clinical wound scores from Day 1 to Day 21 (Mean ± SD).

Day	Control	MTC-U1	p
1	20.86 ± 3.58	20.29 ± 2.56	NS
2	22.00 ± 5.39	18.43 ± 3.15	NS
3	17.00 ± 3.96	14.14 ± 4.18	NS
4	15.14 ± 0.69	13.43 ± 5.62	NS
5	14.43 ± 1.27	12.86 ± 5.55	NS
6	13.00 ± 4.28	9.43 ± 5.32	NS
7	10.86 ± 1.68	6.57 ± 4.12	0.04
9	7.86 ± 1.07	4.43 ± 2.76	0.02
11	6.43 ± 2.70	4.14 ± 3.39	NS
13	3.57 ± 0.98	3.29 ± 2.69	NS
15	2.71 ± 0.49	1.86 ± 1.57	NS
17	0.86 ± 0.38	1.43 ± 0.79	NS
19	0.86 ± 0.38	1.00 ± 0.00	NS
21	0.86 ± 0.38	1.00 ± 0.00	NS

### 3.4 Wound healing dynamics: assessment post-necrotic sequestration

**FIGURE 3:**
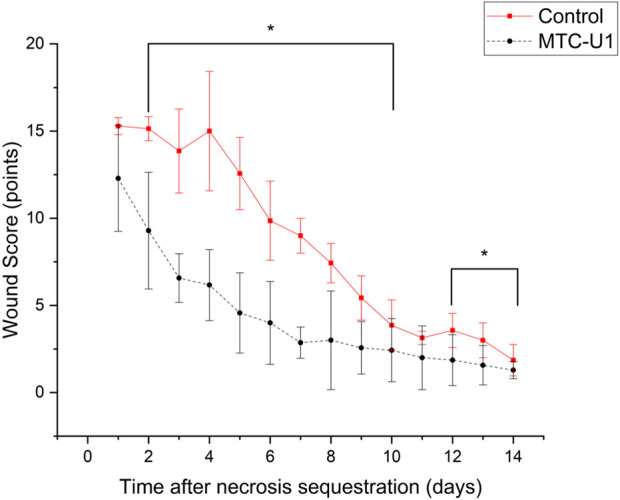
Comparative wound healing kinetics following necrotic tissue sequestration. The line graph illustrates the mean clinical wound scores normalized to the day of complete necrotic detachment (Day 0 post-sequestration). The MTC-U1 formulation (black dashed line) facilitated a significantly faster reduction in wound pathology compared to the Control group (red solid line), achieving a clinically relevant score of <10 points 5 days earlier than the reference therapy. Statistical significance is indicated as *p <* 0.01 (starting from Day 2).

To ascertain the regenerative capacity of the tissue following the acute necrotic phase, the healing trajectory was re-analyzed by normalizing the timeline to the day of complete necrotic tissue detachment (sequestration) for each subject. This analysis demonstrated the superior efficacy of the MTC-U1 formulation compared to the Control group (Figure 3 and Supplementary Material; Supplementary Table S2).

Specifically, wound scores in the MTC-U1 cohort were significantly lower than in the Control group starting as early as Day 2 post-sequestration (*p <* 0.01). This therapeutic advantage was sustained throughout the majority of the subsequent observation period.

A total score of <10 points (indicative of minimal purulent exudate and significant wound contraction) was attained by the MTC-U1 group on Day 1 post-sequestration. In contrast, the Control group did not reach this clinical benchmark until Day 6 post-sequestration. The mean wound healing time from necrotic tissue separation to scab formation is detailed in Supplementary Table S3.

### 3.5 Wound healing duration

**FIGURE 4:**
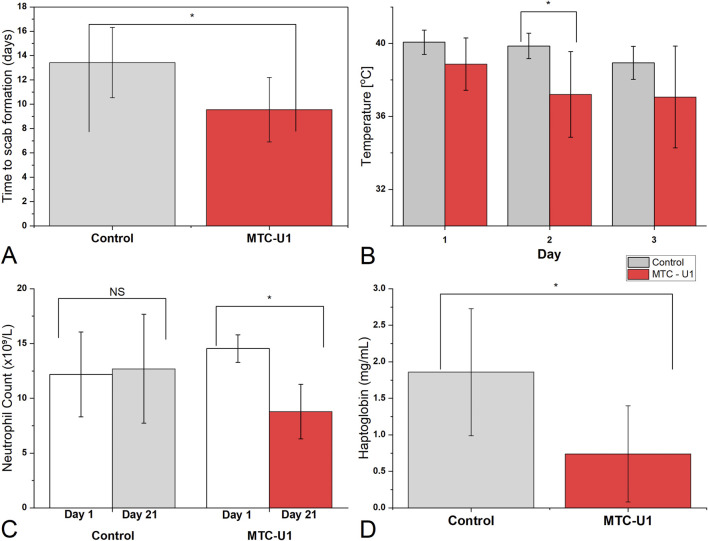
Evaluation of wound healing kinetics and systemic inflammatory markers. **(A)** Time to scab formation (days), representing the period from necrosis sequestration to complete scab formation without exudate. The MTC-U1 group demonstrated a significantly shorter healing period compared to the Control (*p < 0.05). **(B)** Body temperature monitoring (°C) during the initial post-operative phase (Days 1–3). A significant reduction in temperature was observed in the MTC-U1 group on Day 2 compared to the Control (*p < 0.05). **(C)** Peripheral blood neutrophil counts (x10^9^/L) at baseline (Day 1) and study termination (Day 21). The MTC-U1 group showed a significant decrease in neutrophil levels (*p < 0.05), indicating resolution of inflammation, whereas the Control group showed no significant change (NS). **(D)** Comparative analysis of serum haptoglobin levels (mg/mL). Significantly lower concentrations in the MTC-U1 group (*p < 0.05) suggest a mitigated acute-phase response. Data are presented as mean ± SD. Statistical significance is indicated by * (*p <* 0.05); NS = not significant.

The mean healing time, defined as the interval from necrotic tissue sequestration to the formation of a complete scab covering the entire wound without exudate, was analyzed (Figure 4A; Supplementary Table S3). The Control group exhibited a mean duration of 13.43 ± 2.88 days, whereas the MTC-U1 group achieved this endpoint significantly faster, with a mean of 9.57 ± 2.64 days. This finding corresponds to a mean reduction of 3.86 days (*p =* 0.023; 95% CI: 0.64–7.08), representing a substantial effect size (Cohen’s *d =* 1.40).

### 3.6 Qualitative assessment of wound healing mechanisms

**FIGURE 5:**
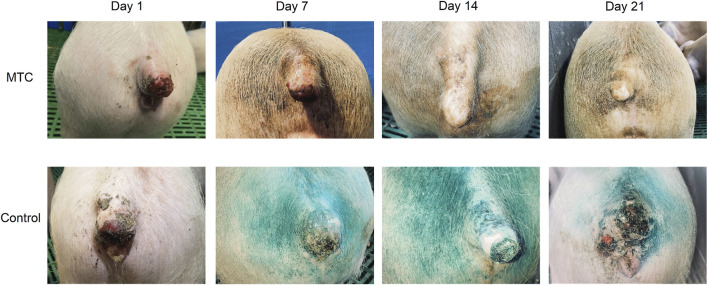
Macroscopic evaluation of wound healing progression. Representative images of the tail necrosis model are shown for the MTC-U1 group (top row) and Control group (bottom row) at Days 1, 7, 14, and 21. The MTC-U1 formulation promoted rapid tissue remodeling with minimal crusting and visible re-epithelialization by Day 14. In contrast, the control group exhibited delayed healing characterized by persistent, rigid scabbing and incomplete wound closure even at Day 21.

Distinct qualitative disparities in the healing trajectory were evident between the study groups (Figure 5; complete photographic documentation for Days 1–21 in Supplementary Table S8). In the MTC-U1 cohort, the therapeutic regimen maintained a sustained moist wound environment throughout the entire treatment period. Consequently, the granulation process proceeded predominantly without crust formation; where present, crusting was minimal and strictly confined to the wound periphery. Clinically, this favorable environment was associated with a markedly reduced volume of seropurulent exudate during the early phase and a complete absence of crust fissuring. In marked contrast, the control group was characterized by the rapid formation of a dry, rigid scab obscuring the entire wound bed. This desiccated scab demonstrated a high degree of vulnerability to mechanical fissuring, resulting in periodic re-exposure of the underlying tissue. Furthermore, the control group exhibited a protracted phase of purulent exudation, which directly contributed to the significantly extended overall healing duration.

### 3.7 Thermographic analysis of inflammation and perfusion

Thermographic assessment was initiated immediately following necrotic tissue sequestration to monitor local inflammatory dynamics. The mean temperature within the ROI at the tail base, serving as a surrogate marker for local inflammation and hyperemia, was significantly reduced in the MTC-U1 group on Day 2 post-sequestration (37.21 °C ± 2.34 °C) compared to the Control group (39.86 °C ± 0.70 °C; *p =* 0.02) (Figure 4B; detailed thermographic data in Supplementary Tables S4–S7). No statistically significant differences were observed on Day 1 (*p =* 0.78) or Day 3.

A parallel trend was evident regarding maximum ROI temperatures, where the difference approached statistical significance on Day 2 (MTC-U1: 38.43 °C ± 2.62 °C vs. Control: 40.60 °C ± 0.50 °C; *p =* 0.09). With regard to distal perfusion, the mean and minimum ROI temperatures of the viable tail tip, indicative of microcirculatory reperfusion, showed no significant intergroup disparities throughout the assessment period, fluctuating within the range of 34.5 °C–36.5 °C.

### 3.8 Hematological and systemic inflammatory profile

A comprehensive overview of hematological and protein parameters is summarized in Table 4 (extended data including temporal dynamics in Supplementary Tables S9–S16). Of particular clinical relevance are the specific temporal dynamics of key inflammatory markers, which evolved over time. On the first day of the study, the mean total leukocyte (WBC) count in both groups exceeded the upper reference limit (22.0 ×10^9^/L), indicating a systemic inflammatory response associated with tissue necrosis. By Day 21, a reduction in WBC was observed in both cohorts, reaching normative physiological values. A pivotal divergence between groups was observed in the dynamics of neutrophil counts (Figure 4C). The MTC-U1 group exhibited a significant resolution of neutrophilia, decreasing from supranormal baseline values (14.54 ± 1.26 ×10^9^/L) to within the reference range (8.79 ± 2.48 ×10^9^/L; *p <* 0.05 for Day 1 vs. Day 21). Conversely, the Control group showed no resolution; neutrophil counts persisted above the upper limit (10.0 ×10^9^/L) and even exhibited a slight, non-significant increase from 12.18 ± 3.88 to 12.70 ± 4.97 ×10^9^/L (*p >* 0.05). Monocyte counts declined significantly in both groups between Day 1 and Day 21 (Control: 2.28 ± 0.21 to 0.59 ± 0.09 ×10^9^/L, *p <* 0.01; MTC-U1: 2.89 ± 1.02 to 0.97 ± 0.22 ×10^9^/L, *p <* 0.01). Baseline values in both groups exceeded the upper reference limit (2.2 ×10^9^/L), a finding consistent with the demand for phagocytic clearance of necrotic tissue during the initial phase. With respect to systemic inflammation markers, serum haptoglobin concentration on Day 21 was significantly higher in the Control group (1.86 ± 0.87 mg/mL) compared to the MTC-U1 group (0.74 ± 0.66 mg/mL; *p <* 0.05) (Figure 4D). This elevation in the Control group suggests a sustained acute-phase response, whereas the MTC-U1 group values returned to levels closer to the physiological norm (∼1.0 mg/mL). A subsequent analysis of fibrinogen levels revealed no statistically significant intergroup disparities (Control: 4.04 ± 0.25 g/L vs. MTC-U1: 4.76 ± 0.92 g/L).

**TABLE 4:** Hematological and protein parameters on Days 1 and 21 (Mean ± SD).

Parameter	Day	Control	±SD	MTC-U1	±SD	Ref. Range	p (Control vs. MTC-U1)
HEMATOLOGY							
WBC [×10^9^/L]	1	27.98	1.27	28.48	3.48	11–22	NS
WBC [×10^9^/L]	21	21.61	5.73	23.31	3.39	11–22	NS
Neutrophils [×10^9^/L]	1	12.18	3.88	14.54	1.26	3–10	NS
Neutrophils [×10^9^/L]	21	12.70	4.97	8.79	2.48	3–10	*p <* 0.01
Neutrophils [%]	1	45.53	11.13	53.73	0.60	28–47	NS
Neutrophils [%]	21	47.95	9.82	37.11	7.05	28–47	*p <* 0.05
Lymphocytes [×10^9^/L]	1	12.49	2.72	8.96	2.36	4.2–13.6	*p <* 0.05
Lymphocytes [×10^9^/L]	21	10.15	1.00	12.36	1.03	4.2–13.6	*p <* 0.01
Lymphocytes [%]	1	42.43	9.76	29.53	2.71	39–62	*p <* 0.05
Lymphocytes [%]	21	45.97	9.72	53.48	7.86	39–62	NS
Monocytes [×10^9^/L]	1	2.28	0.21	2.89	1.02	0.2–2.2	NS
Monocytes [×10^9^/L]	21	0.59	0.09	0.97	0.22	0.2–2.2	*p <* 0.01
Monocytes [%]	1	8.21	0.66	9.33	1.85	2–10	NS
Monocytes [%]	21	2.72	0.47	3.99	0.84	2–10	*p <* 0.05
Eosinophils [×10^9^/L]	1	0.52	0.08	1.66	0.51	0.5–2.4	*p <* 0.05
Eosinophils [×10^9^/L]	21	0.61	0.19	0.98	0.36	0.5–2.4	*p <* 0.05
Eosinophils [%]	1	1.80	0.24	5.92	1.49	0.5–11	*p <* 0.01
Eosinophils [%]	21	2.55	0.41	4.45	0.83	0.5–11	*p <* 0.01
Basophils [×10^9^/L]	1	0.28	0.09	0.41	0.07	0–0.4	*p <* 0.05
Basophils [×10^9^/L]	21	0.19	0.10	0.22	0.06	0–0.4	NS
Basophils [%]	1	0.96	0.29	1.48	0.28	0–2	*p <* 0.01
Basophils [%]	21	0.81	0.31	0.88	0.26	0–2	NS
PROTEIN PROFILE (Day 21)							
Total protein [g/L]	21	64.23	3.76	64.66	5.01	35–60	NS
Albumin [g/L]	21	23.61	2.96	20.36	2.52	19–24	NS
Globulin [g/L]	21	40.61	4.68	44.30	6.76	​	NS
A/G ratio	21	0.59	0.12	0.48	0.13	0.59–1.13	NS
ACUTE PHASE PROTEINS (Day 21)							
Haptoglobin [mg/mL]	21	1.86	0.87	0.74	0.66	≤1.0	*p <* 0.05
Fibrinogen [g/L]	21	4.04	0.25	4.76	0.92	1–5	NS

### 3.9 Microbiological analysis

A subsequent microbiological evaluation of the tail wounds revealed a predominance of opportunistic microbiota. The isolates predominantly comprised *Escherichia coli* (indicative of fecal contamination inherent to the porcine housing environment), coagulase-negative *Staphylococcus* spp. (commensal skin flora), and *Streptococcus* canis (oral microbiota). The investigation revealed that all isolated strains demonstrated low pathogenic potential and retained susceptibility to standard veterinary antibiotics. No statistically significant intergroup disparities were observed regarding the spectrum of isolated microorganisms.

### 3.10 Safety and tolerability assessment

No signs of dermal irritation were observed following the topical application of MTC-U1 throughout the entire study duration. Specifically, the treatment site showed no evidence of erythema, edema, cutaneous lesions, pruritus, or increased tenderness. Furthermore, contrary to inducing local inflammation, thermographic data indicated a reduction in skin surface temperature. The mean irritation score (*x*_*av*_) remained below 0.5, classifying the formulation as non-irritating in accordance with the ISO 10993-10 standard. Notably, a skin-smoothing effect, characterized by reduced roughness of the stratum corneum, was observed at the application site during the terminal phase of the study. The systemic clinical status of the animals in the MTC-U1 group remained stable and satisfactory throughout the experiment. Core body temperature was maintained at 38.6 °C ± 0.4 °C with no significant fluctuations, and no adjunctive medical interventions were necessary.

### 3.11 Analysis of production parameters and body weight dynamics

Analysis of Covariance (ANCOVA) demonstrated a statistically significant effect of the MTC-U1 treatment on final body weight (*F* (1,11) = 6.75; *p =* 0.025; ηp^2^ = 0.38, indicating a large effect according to Cohen’s conventions where ηp^2^ ≥ 0.14 represents a large effect) after adjusting for baseline body weight as a covariate. The Estimated Marginal Means (EMM) for the final body weight were significantly higher in the MTC-U1 group (42.68 kg; 95% CI: 39.84–45.51) compared to the Control group (37.94 kg; 95% CI: 35.10–40.78). Furthermore, the Feed Conversion Ratio (FCR), evaluated here as a composite index of general health status and animal welfare, was more favorable in the MTC-U1 group compared to the control group (Table 5; detailed production data in Supplementary Tables S17, S18). The superior FCR observed in the treated cohort suggests a reduced metabolic burden associated with the inflammatory response, thereby facilitating accelerated convalescence and tissue repair.

**TABLE 5:** Production parameters.

Parameter	Control	MTC-U1
Initial body weight (kg)	25.32 ± 2.16	31.46 ± 6.26
Final body weight (kg)	34.91 ± 4.58	45.71 ± 6.01
Total weight gain (kg/21 days)	9.59 ± 5.51	14.26 ± 0.64
ADG (g/day)	456.67	678.91
FCR	1.69	1.44

In summary, MTC-U1 treatment resulted in a significant acceleration of wound healing (a reduction of 3.86 days), a reduction of local and systemic inflammation (as measured by thermography, haptoglobin, and neutrophils), and an improvement in production parameters compared to standard oxytetracycline therapy, with an excellent local tolerance profile.

## 4 Discussion

The findings of this investigation demonstrate that the MTC-U1 ointment, formulated with a multifunctional patented carrier incorporating 3.0% (w/w) *Curcuma longa* extract and 1.6% (w/w) berberine hydrochloride, significantly accelerates the healing of complex chronic wounds in a translational porcine model compared to standard antibiotic therapy. The observed reduction in healing time by 3.86 days (corresponding to a 29% decrease) is of profound clinical relevance. In the context of chronic wound management, delayed closure of a wound has been shown to be associated with an increased risk of infectious complications, an amplified systemic metabolic burden, and escalated healthcare costs (Sen, 2021).

The MTC-U1 system occupies a distinct niche within the emerging landscape of advanced wound healing delivery platforms. Recent studies have demonstrated the efficacy of biogenic nanosilver-based acticoat systems employing tri-modal evaluation (*in silico, in vitro, and in vivo*) for infected wounds (Singh et al., 2023), as well as multifaceted green-synthesized silver-alginate-ciprofloxacin hydrogels designed for complex wound environments (Singh et al., 2026). While these systems rely on metallic nanoparticles or synthetic antibiotics as primary antimicrobial agents, the MTC-U1 approach harnesses plant-derived bioactives (curcumin and berberine) delivered through a chemically defined penetration enhancement mechanism. This distinction is clinically significant in the context of growing regulatory scrutiny over nanoparticle cytotoxicity and environmental persistence, and supports the broader trend toward phytochemical-based alternatives in antimicrobial wound care (Moses et al., 2023).

The MTC-U1 formulation demonstrated a highly favorable safety profile. The complete absence of irritation reactions (x_av_ < 0.5) is particularly noteworthy given the inclusion of DMSO, a permeation enhancer known to induce cutaneous irritation at elevated concentrations. However, the specific concentration of DMSO utilized in the MTC carrier falls well within the range established as safe for topical administration (Williams and Barry, 2004; Schafer et al., 2023). Our findings are also consistent with very recent data concerning other flavonoid-rich extracts. For example*, Retama raetam* extract was shown to enhance wound contraction and re-epithelialization through similar anti-inflammatory pathways (Bouzenna et al., 2025). However, in contrast to the numerous ethnomedicinal studies that employ only crude extracts, our approach utilizes a sophisticated carrier (MTC). This delivery system ensures that the active moieties, curcumin and berberine, effectively penetrate the thickened eschar of chronic porcine wounds, offering a distinct translational advantage.

The accelerated healing trajectory observed in the MTC-U1 group results from the synergistic interplay between the specialized carrier system and the incorporated active moieties. *Ex vivo* characterization of the vehicle (Figure 2) revealed that the MTC-U(Na) carrier induces progressive structural modifications within the skin barrier—a 72% increase in stratum corneum thickness at 3 h (indicative of hydration and swelling) and a 3.2-fold enhancement in membrane permeability at 12 h (reflecting lipid reorganization and enhanced propidium iodide penetration) — creating transcellular and paracellular channels that facilitate the dermal delivery of both curcumin (MW 368 Da, log P 3.2) and berberine (MW 336 Da, log P 3.6). These *ex vivo* observations provide a direct mechanistic framework for the rapid *in vivo* pharmacological response: the significant reduction in local inflammatory temperature observed as early as Day 2 post-application (37.2 °C vs. 39.9 °C; *p* < 0.05; Figure 4) indicates that therapeutically relevant concentrations of the active agents reached the wound bed within 24–48 h of initial application. Furthermore, the systemic normalization of neutrophil counts and haptoglobin levels in the MTC-U1 group provides indirect pharmacokinetic evidence that berberine, in particular, achieved sufficient dermal-to-systemic absorption to modulate the acute-phase response. The effects of these substances are further amplified by the presence of DMSO and urea, consistent with their well-established roles as permeation enhancers (Schafer et al., 2023). DMSO reversibly disrupts the ordered lipid lamellae of the stratum corneum by intercalating between ceramide head groups and displacing bound water molecules (Williams and Barry, 2004). Concurrently, urea destabilizes keratin secondary structure through hydrogen bond competition and promotes corneocyte dissociation, effectively reducing the diffusional path length for permeating molecules (Schafer et al., 2023). When combined, these mechanisms operate on complementary structural domains (intercellular lipids vs. corneocyte protein matrix), yielding enhancement factors that exceed the sum of individual contributions. Furthermore, the incorporation of a water-soluble curcumin extract (curcuminoid content >20%) represents a deliberate formulation strategy to maximize the aqueous-phase concentration gradient driving passive diffusion, circumventing the dissolution-limited absorption characteristic of native crystalline curcumin. Crucially, while these permeation kinetics were established on intact skin barriers, the compromised epidermal integrity characteristic of chronic wounds likely facilitates even greater bioavailability of the active ingredients. The MTC platform offers several advantages over alternative delivery systems investigated for curcumin in wound healing applications. Nanoparticle formulations, while effective in enhancing curcumin solubility, suffer from physical instability during storage and limited penetration through the necrotic tissue layer of chronic wounds (Krausz et al., 2015). Hydrogel systems, although providing excellent moisture retention, typically lack active penetration enhancement mechanisms. In contrast, the MTC system integrates three complementary functionalities: (1) active permeation enhancement via DMSO/urea synergy, (2) sustained release from the lipophilic vehicle matrix (porcine lard, goose fat, eucerin), and (3) wound bed conditioning through occlusion-mediated hydration. This tripartite mechanism is reflected in the clinical outcomes, where the MTC-U1 group demonstrated not only accelerated healing but also qualitatively superior granulation tissue formation (Figure 5), suggesting that effective delivery of curcumin and berberine to the proliferating wound margin was achieved throughout the treatment period. This hypothesis is corroborated by the early thermographic normalization discussed above (Figure 4), confirming swift penetration and immediate local pharmacological action of the active moieties at the wound site. Within the tissue, these agents exert complementary anti-inflammatory effects via distinct molecular pathways. Curcumin is known to inhibit NF-κB activation, downregulate COX-2 expression, and suppress TNF-α production (Panchatcharam et al., 2006; Akbik et al., 2014; Prasad et al., 2014; Kandaswamy et al., 2024). In the present study, this pharmacological activity is clinically mirrored by the resolution of neutrophilia and the significant reduction in serum haptoglobin levels (detailed in the hematological analysis below). Despite the lack of direct measurement of NF-κB activity or COX-2 expression in this study, the observed normalization of systemic inflammatory markers is consistent with the anti-inflammatory mechanisms attributed to curcumin in the extant literature. Berberine, conversely, targets MAPK and Akt signaling pathways and suppresses the secretion of pro-inflammatory cytokines (IL-1β, TNF-α, IL-6), while simultaneously providing direct antimicrobial activity against common wound pathogens (Neag et al., 2018; Li et al., 2023). In the context of our study, the specific contribution of berberine to the observed clinical outcomes cannot be isolated from that of curcumin or the carrier system; this represents a limitation inherent to multi-component formulation testing. The lipid-based carrier, distinct from its delivery function, maintains a moist wound microenvironment via occlusion, thereby preventing premature crust formation and optimizing keratinocyte migration. This physical effect is corroborated by macroscopic evaluation (Figure 5), where MTC-U1 treated wounds displayed a granulation tissue structure distinctly different from the rigid scabs observed in the Control group. Consequently, the 29% reduction in healing time (3.86 days; *p =* 0.023; 95% CI: 0.64–7.08) represents the cumulative benefit of this multimodal mechanism: a delivery system that enhances the bioavailability of anti-inflammatory agents while simultaneously optimizing the physiology of the wound bed through occlusion. Finally, ANCOVA modeling confirmed that the observed body weight gain was a direct anabolic effect of the MTC-U1 treatment, independent of baseline weight variations. The magnitude of this effect (ηp^2^=0.38) is substantial, effectively ruling out selection bias as a potential confounding factor.

The hematological profile provides substantial evidence that supports the carrier-mediated enhancement of bioavailability. The rapid normalisation of systemic inflammatory markers within the first week of treatment constitutes indirect pharmacokinetic evidence that curcumin and berberine achieved therapeutically relevant concentrations within the wound tissue, translating into measurable reductions in serum haptoglobin and neutrophil levels. In large animal models, the resolution of local tissue inflammation is strongly correlated with the normalization of acute-phase proteins. The significant reduction in serum haptoglobin and neutrophil counts in the MTC-U1 group, observed in the absence of systemic antibiotic therapy, suggests that the topical application effectively controlled the local infection-inflammation axis, preventing the spillover of inflammatory mediators into the systemic circulation, a key goal in the management of chronic, infected wounds. Specifically, the resolution of neutrophilia in the MTC-U1 group (decreasing from 14.54 to 8.79 ×10^9^/L) stands in marked contrast to the Control group, where no improvement was observed (increase from 12.18 to 12.70 ×10^9^/L). Since neutrophilia is a hallmark response to bacterial wound infection (Petersen et al., 2004), its resolution correlates directly with the clinical improvement of the wound status. Of particular significance is the disparity in haptoglobin levels, recognized as one of the most sensitive acute-phase proteins in porcine models (Petersen et al., 2004). The significantly lower concentration in the MTC-U1 group (0.74 vs. 1.86 mg/mL; *p <* 0.05) confirms that local therapeutic application translates into effective modulation of the systemic inflammatory response. This immunomodulatory capability is critical for diabetic and chronic ulcers, where the pro-inflammatory M1 macrophage phenotype often persists unnaturally. Recent investigations into curcumin delivery systems have further validated that controlled release of curcumin effectively shifts the macrophage population towards the pro-regenerative M2 phenotype, thereby reducing oxidative stress and promoting collagen deposition (Wójcik et al., 2023). In the context of our study, the rapid normalization of neutrophil counts and haptoglobin levels serves as a macroscopic proxy for this underlying microscopic resolution.

It is noteworthy that wounds treated with the MTC-U1 preparation exhibited a persistent moist microenvironment, accompanied by minimal to no scab formation during granulation. This observation aligns with the established paradigm of moist wound healing, initially proposed by Winter (1962) using a porcine model (Winter, 1962). A hydrated environment facilitates keratinocyte migration, fibroblast proliferation, and granulation tissue formation, whereas a desiccated scab acts as a mechanical impediment to these processes (Junker et al., 2013). In contrast, the control group demonstrated accelerated scab formation, accompanied by a propensity for fissuring. This phenomenon led to recurrent wound exposure and protracted healing periods. The lipid vehicle utilized in this study, composed of porcine and goose lard, eucerin, and other ingredients, functions to occlude the skin, thereby retaining moisture while facilitating gas exchange. Additionally, the zinc sulfate component may promote epithelialization by functioning as a cofactor for the matrix metalloproteinases (MMPs) critical to tissue remodeling (Lansdown et al., 2007).

The selection of ‘time to complete scab formation’ rather than ‘time to 100% re-epithelialization’ as the primary endpoint warrants explicit justification. In the context of Grade 4 tail-biting injuries in production animals, serial biopsies for histological verification of epidermal continuity would require repeated invasive procedures under general anesthesia, a protocol that was contraindicated by the Ethics Committee on animal welfare grounds. Nevertheless, the convergence of clinical scoring, thermographic normalization, and resolution of systemic inflammatory markers (neutrophil counts and haptoglobin) provides multi-modal corroborative evidence that the observed scab formation reflects functional tissue repair. Future studies should incorporate terminal histopathological analysis employing H&E and Masson’s Trichrome staining to verify epidermal continuity, collagen deposition, and the type I/III collagen ratio beneath the eschar, thereby providing definitive morphological validation of healing quality.

Consequently, the porcine tail wound model utilized in the present study is posited to hold high translational validity for several reasons. Firstly, porcine skin exhibits the highest degree of structural homology to human integument among standard animal models, featuring comparable epidermal thickness, hair follicle density, and microvascular architecture (Sullivan et al., 2001; Seaton et al., 2015; Elloso et al., 2025). Secondly, tail lesions resulting from biting constitute a representative model of complex chronic wounds; they combine characteristics of lacerations, puncture wounds, and contusions with associated necrosis, inflammation, and secondary infection, thereby mimicking numerous clinical scenarios encountered in human medicine (Taylor et al., 2010). Thirdly, this model carries intrinsic applicative significance in veterinary medicine, as tail biting represents a critical welfare issue in swine production that generates substantial economic losses (D’Eath et al., 2014).

To date, a limited number of studies have evaluated curcumin formulations utilizing a porcine wound model. Sidhu et al. (1998) demonstrated accelerated healing of acute wounds in rats following topical curcumin application; however, the translational relevance of the rodent model is constrained by significant anatomical and physiological cutaneous disparities compared to humans. Mohanty et al. (2012) validated the efficacy of a curcumin oleogel in a chronic wound model using diabetic mice, evidencing enhanced re-epithelialization and angiogenesis. Furthermore, Singer et al. (2007) showed that oral administration of curcumin in rats effectively mitigates necrotic progression within the ischemic zone of burn wounds, an effect attributed to its capacity for free radical scavenging and reduction of oxidative stress. In turn, Krausz et al. (2015) established in a murine model that nanoparticle-encapsulated curcumin not only accelerates wound healing by stimulating neovascularization and collagen deposition but also exhibits potent antimicrobial activity against MRSA and *P. aeruginosa* infections. The present study, which was conducted using a highly translational porcine model, provides robust evidence supporting the clinical application of curcumin in the management of chronic wounds.

The present findings should be interpreted within the context of wound healing strategies assessed in animal models. In a Wistar albino rat burn model, the topical application of Ankaferd Blood Stopper (ABS) facilitated the healing process, showing efficacy comparable to silver sulfadiazine regarding re-epithelialization and inflammation reduction (Şensoy et al., 2024). Additionally, a current comprehensive review of polymeric wound dressings discusses the utility of various hydrogel and non-hydrogel systems in wound management, emphasizing the functional properties of natural and synthetic polymers (Zamani et al., 2026). Notably, recent investigations into advanced biomaterials have highlighted the efficacy of micro-nano architectured scaffolds. For instance, a study on silica fiber scaffolds demonstrated that synergistic structural features can significantly accelerate wound healing processes, offering a promising alternative to traditional hydrogel-based systems (Christ et al., 2026). These findings suggest that optimizing the physical architecture of wound dressings is as critical as their chemical composition in promoting tissue regeneration. The porcine model employed in the present study offers a closer approximation to human skin biology, thereby providing a more robust preclinical evidence base for subsequent clinical translation.

The present study is subject to several limitations that warrant consideration when interpreting the results. First, the sample size of seven animals per group, while sufficient to detect large effect sizes, represents a constraint inherent to translational research involving large animal models. A *post hoc* power analysis revealed that for the primary endpoint (healing time), given the observed effect size (Cohen’s *d* = 1.39) and a pooled standard deviation of 2.76 days, the achieved statistical power was 0.67. This value falls below the conventional threshold of 0.80; however, the substantial effect size permitted the detection of a statistically significant difference between groups. Similarly, regarding the baseline body weight discrepancy between groups (Control: 25.32 ± 2.16 kg vs. MTC-U1: 31.46 ± 6.26 kg; pooled SD = 4.68 kg; Cohen’s *d* = 1.31), *post hoc* power analysis yielded a value of 0.62. Suboptimal statistical power in both instances necessitates a cautious interpretation of the findings and underscores the need for confirmatory studies in a larger cohort. Crucially, however, ANCOVA confirmed the independence of the treatment effect on final body weight from baseline differences (F (1,11) = 6.75; *p =* 0.025), and baseline wound severity scores did not differ significantly between groups (*p =* 0.88). These findings indicate that the body weight imbalance did not confound the primary healing outcome.

Complete blinding of observers was precluded by the distinct physical forms of the treatments (ointment vs. spray). To mitigate potential detection bias, photographic documentation was anonymized and adjudicated retrospectively by two independent investigators who were blinded to treatment allocation. Furthermore, the primary inflammatory endpoints (thermographic temperature, neutrophil count, haptoglobin) represent objective measurements not susceptible to observer bias. Nevertheless, the possibility of unconscious bias during daily clinical evaluations by unblinded personnel constitutes a recognized limitation of this study and should be addressed through the use of identical vehicle forms (e.g., placebo ointment vs. active ointment) in future confirmatory trials. The use of oxytetracycline as an active comparator, rather than a placebo or vehicle-only control, constitutes a critical limitation of this study. As demonstrated in the *ex vivo* characterisation (Section 3.1), the MTC carrier itself induces measurable structural modifications of the skin barrier (stratum corneum thickening and enhanced membrane permeability). These carrier-mediated effects, encompassing occlusion, hydration, and lipid reorganisation, may independently contribute to the observed acceleration of wound healing. Consequently, the 29% reduction in healing time should be interpreted as a formulation-level outcome of the complete MTC-U1 system, rather than being exclusively attributed to the pharmacological synergism of curcumin and berberine. The ethical constraints of the approved protocol (Resolution No. 18/2020), which precluded leaving Grade 4 chronic infected wounds without pharmacologically active treatment, prevented the inclusion of a vehicle-only arm. Future studies should incorporate a carrier-only control group, ideally using a sham-wound or lower-grade lesion model where ethical approval for a placebo arm may be feasible, to definitively dissect the relative contributions of the carrier vehicle and the active phytochemicals.

Finally, the microbiological assessment employed a qualitative culture-based approach, which, while sufficient for pathogen identification and susceptibility profiling, does not permit quantitative determination of bacterial load reduction (log CFU). Future studies should incorporate serial quantitative wound cultures with standardized biopsy sampling and, ideally, qPCR-based biofilm matrix quantification to rigorously evaluate the antimicrobial efficacy of the MTC-U1 formulation.

The proprietary nature of the MTC-U1 formulation, with precise excipient ratios protected by patents (PL245223B1; US12478679B2), limits direct reproducibility by independent researchers. However, the fundamental composition, functional characteristics, and mechanism of action are disclosed in the cited patents and this manuscript, enabling the evaluation of the therapeutic rationale and design analogous formulations for independent validation.

The findings of the present study warrant further investigation into the utility of the MTC-U1 formulation for the management of hard-to-heal wounds and delineate several priority avenues for future development. Primarily, placebo-controlled studies with full blinding in a larger animal cohort are recommended. This approach would enhance statistical power and eliminate potential systematic biases associated with unblinded observation. Concurrently, a comprehensive histopathological evaluation employing immunohistochemical analysis is essential. This should encompass markers of angiogenesis, myofibroblast differentiation, and cell proliferation, alongside the type I/III collagen ratio, to objectively validate the quality of the regenerated tissue. Elucidating the underlying mechanism of action of MTC-U1 requires molecular-level investigations, specifically analyzing the expression profiles of key pro- and anti-inflammatory cytokines (IL-1β, IL-6, IL-10, TNF-α) as well as growth factors pivotal to the healing process (TGF-β, VEGF, EGF, FGF). Furthermore, formulation optimization is advisable through the systematic determination of optimal curcumin and berberine hydrochloride concentrations. This approach aims to maximize therapeutic efficacy while maintaining a favorable safety profile.

The high translational validity of the porcine model, as demonstrated in this study, supports the initiation of clinical trials in human patients with chronic wounds. Venous leg ulcers, diabetic foot syndrome, surgical wounds with impaired healing, and pressure ulcers emerge as particularly promising target indications, especially in immobilized patients where these conditions represent a significant clinical challenge. In each of these contexts, the synergistic combination of anti-inflammatory properties, granulation support, and the maintenance of a moist wound environment may yield substantial therapeutic benefits.

## 5 Conclusions

This controlled preclinical study demonstrates that MTC-U1 ointment containing *Curcuma longa* extract (3% w/w) and berberine hydrochloride (1.6% w/w) in a multifunctional carrier significantly accelerates wound healing in a translational porcine model of chronic infected tail-biting injuries. The initial study hypotheses were confirmed. The MTC-U1 formulation attenuated both local and systemic inflammation, as evidenced by thermographic and hematological assessments. It reduced healing time by 3.86 days compared to standard oxytetracycline therapy and promoted moist wound healing with minimal crust (scab) formation. The excellent local tolerance profile observed despite the inclusion of DMSO supports the safety of this delivery system. These findings provide preclinical evidence for MTC-U1 as a potential antibiotic-sparing alternative in chronic wound management and warrant further studies incorporating histopathological validation, followed by clinical trials in patients with hard-to-heal wounds.

## Data Availability

The original contributions presented in the study are included in the article/Supplementary Material, further inquiries can be directed to the corresponding author.
