# Supplementary material for: Curcumin-berberine ointment based on multifunctional carrier accelerates wound healing in a translational porcine model of tail-biting injuries: a controlled study against oxytetracycline
Source: Front Pharmacol. 2026 Apr 8;17:1798208. doi: 10.3389/fphar.2026.1798208 (PMC13099298; doi:10.3389/fphar.2026.1798208)
Supplement: Supplementary file 1 [file DataSheet1.pdf]

Supplementary Materials

Curcumin-Berberine Ointment Based on Multifunctional Transdermal Carrier Accelerates Wound Healing in a Translational Porcine Model of Tail-Biting Injuries: A Controlled Study Against Oxytetracycline

Paweł Biernat (0000-0002-5308-4426)[1,2], Dominik Marciniak (0000-0002-3326-0840)[1,2], Dawid Bursy (0000-0002-5729-6717)[1,2], Konrad Krajewski (0000-0001-7518-4228)[2,3], Radosław Balwierz[✉] (0000-0002-6173-2702)[2,4]

1 - Department of Drug Forms Technology, Faculty of Pharmacy, Wrocław Medical University, 211 Borowska St. Wroclaw

2 - Biotts SA, Wroclawska 44c St., 55-040 Bielany Wroclawskie, Poland

3 - Wroclaw University of Science and Technology, Faculty of Computer Science and Management,

4 - Institute of Chemistry, University of Opole, Oleska 48 St. 45-052 Opole, Poland

✉ **Correspondence:**

- Radoslaw Balwierz, Institute of Chemistry, University of Opole, Oleska St. 48, 45-052 Opole, Poland, email: radoslaw.balwierz@uni.opole.pl

Table of content

Ex vivo analysis 3

Fig. S1. A fragment of the skin from which skin trepane samples were taken. Visible holes are biopsy sites (arrow). 3

Table S1. Biopsies of piglets for each group – MTC-U, MTC-U(Na) and MTC-U(A) 4

Fig. S2. Graph of the thickness of the keratinized layer of epidermis with standard deviation for MTC-U 5

Fig. S3. Graph of the thickness of reproductive layer of epidermis with standard deviation for MTC-U 5

Fig.S4. Graph of the thickness of the keratinized layer of epidermis with standard deviation for MTC-U(Na) 6

Fig. S5. Graph of the thickness of reproductive layer of epidermis with standard deviation for MTC-U(Na) 6

Fig. S6. Graph of the thickness of the keratinized layer of epidermis with standard deviation for MTC-U(A) 7

Fig. S7. Graph of the thickness of reproductive layer of epidermis with standard deviation deviation for MTC-U(A) 7

In vivo analysis 8

Table. S2. Wound healing based on the scoring in individual groups evaluated from the day of separation of necrotic tissue 8

Table S3. Mean wound healing time counted from the day of separation of necrotic tissue to the formation of scab (assessment of the size of 1/5th of the wound), without the occurrence of swelling, redness, purulent leakage and serous exudate. 8

Table S4. Thermographic evaluation: Mean temperature of ROI of the tail base area in individual groups from the day of separation of necrotic tissue 8

Table S5. Thermographic evaluation: maximum ROI temperature of the tail base area in individual groups on the first 3 days after separation of necrotic tissue 9

Table S6. Thermographic evaluation of wound healing: mean temperature of tail tip ROI in individual groups on the first 3 days after separation of necrotic tissue 9

Table S7. Thermographic evaluation of wound healing: minimum tail tip ROI temperature in individual groups on the first 3 days after separation of necrotic tissue 9

Table S8. Macroscopic evaluation of wound healing progression from day 1 to day 21 10

Table S9. Mean number of leukocytes in examined animals in particular groups depending on the time of intake. 24

Table S10. Relative and absolute number of neutrophils in tested animals in individual groups depending on the time of intake. 24

Table S11. Relative and absolute number of lymphocytes in examined animals in particular groups depending on the time of intake. 24

Table S12. Relative and absolute number of monocytes in experimental groups according to the sampling time. 24

Table S13. Relative and absolute number of acid-absorbent granulocytes (eosinophils) in experimental groups according to the sampling time. 25

Table S14. Relative and absolute number of alkaline granulocytes (basophils) in tested animals in particular groups depending on the time of collection. 25

Table S15. Protein parameters as indicators of inflammation in examined animals in particular groups 25

Table S16. Mean concentrations of acute phase proteins (haptoglobin and fibrinogen) in the examined groups of animals. 25

Table S17. Weight production indicators 26

Table S18. Basic production indicators: daily increments, mean daily feed intake and feed conversion rate. 26

Ex vivo analysis

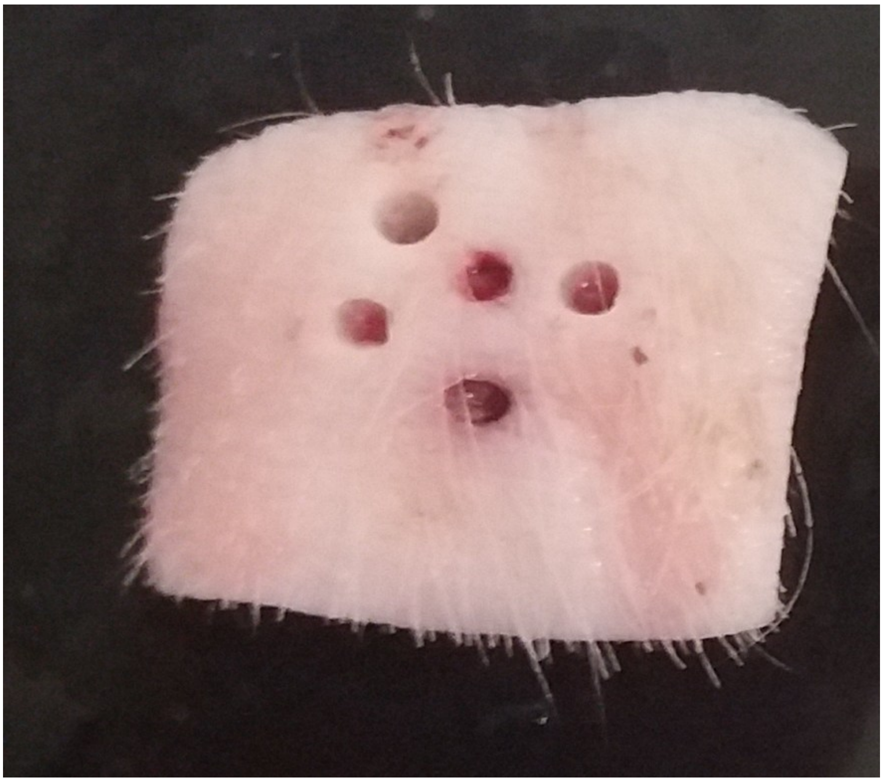

Fig. S1. A fragment of the skin from which skin trepane samples were taken. Visible holes are biopsy sites (arrow).

Table S1. Biopsies of piglets for each group – MTC-U, MTC-U(Na) and MTC-U(A)

	0 min	1 h	3 h	6 h	12 h
MTC-U0	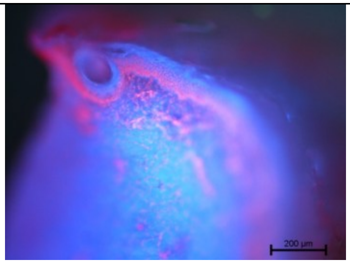	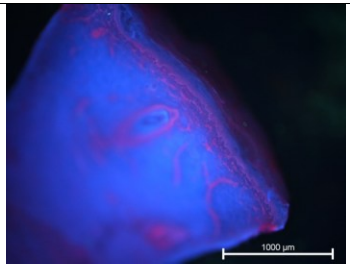	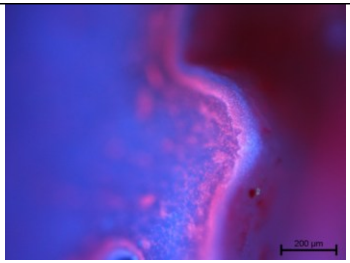	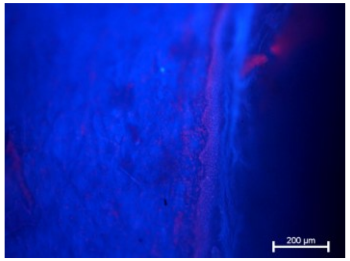	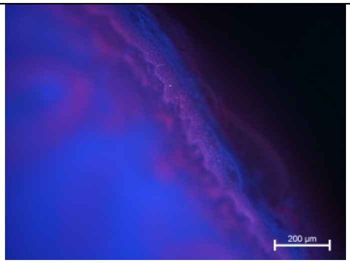
MTC-U(Na)	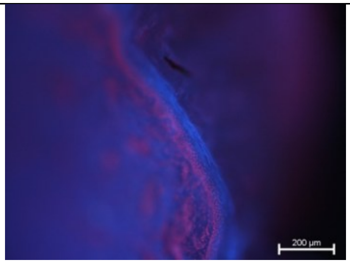	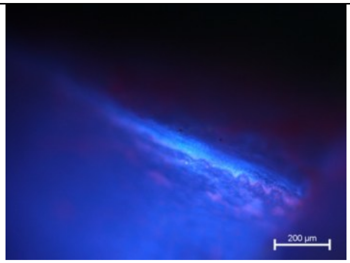	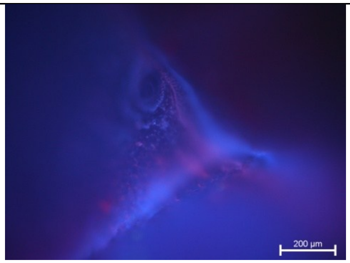	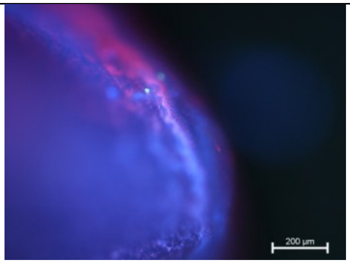	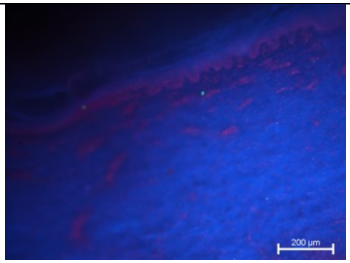
MTC-U(A)	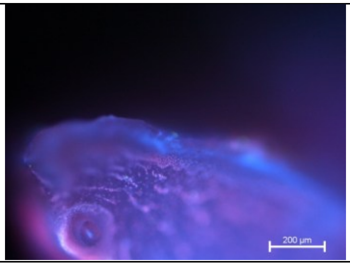	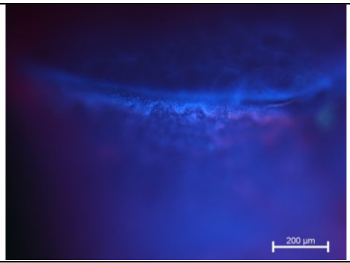	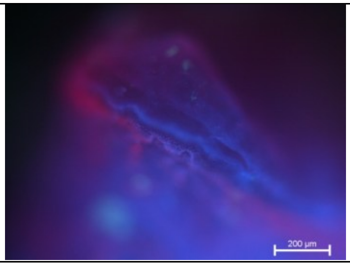	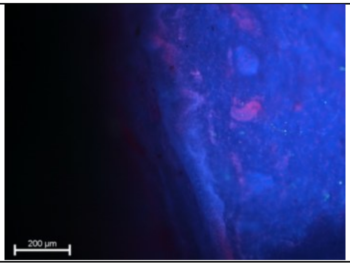	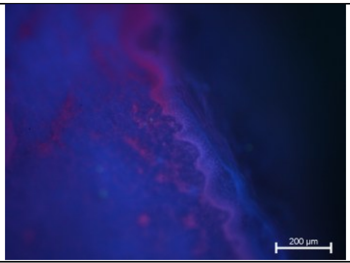

NOTE:

Propidium iodide and DAPI are chemical compounds that belong to the group of fluorescent dyes (fluorochromes), used in histology primarily to detect nucleic acids. DAPI and more precisely 4 ', 6-diamidino-2-phenylindole due to its properties penetrates inside the cells, there it binds to deoxyribonucleic acid (DNA) and when excited with light from the range of near ultraviolet it shines blue. In contrast to propidium iodide (PI), it also has the ability to penetrate intact cell membrane, and can therefore be used to stain live cells. In turn, propidium iodide only penetrates inside those cell organelles that have been damaged, causing red coloration of the nucleic acids contained in them (Kapuscinski, 1995; Jędrzejowska and Kubrakiewicz, 2010)

REF:

Kapuscinski J. DAP; a DNA-Specific Fluorescent Probe. Biotechnic and Histochemistry, 70 (5): 220–233,1995. https://doi.org/10.3109/10520299509108199.

Jędrzejowska I. i Kubrakiewicz J. Yolk nucleus - The complex assemblage of cytoskeleton and ER is a site of lipid droplet formation in spider oocytes. Arthropod Structure and Development, 39(5): 350–359, 2010. https://doi.org/10.1016/j.asd.2010.05.001.

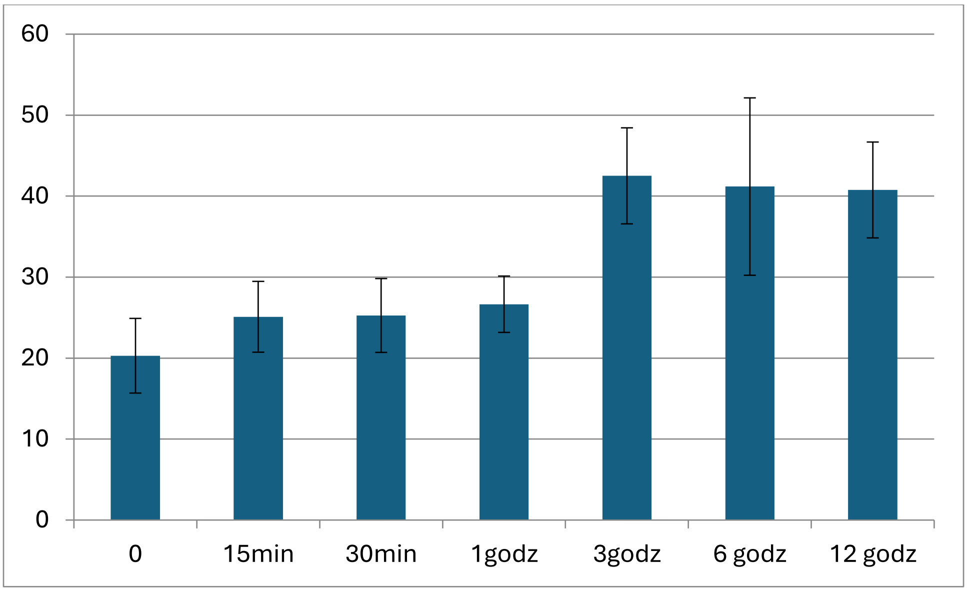

Fig. S2. Graph of the thickness of the keratinized layer of epidermis with standard deviation for MTC-U

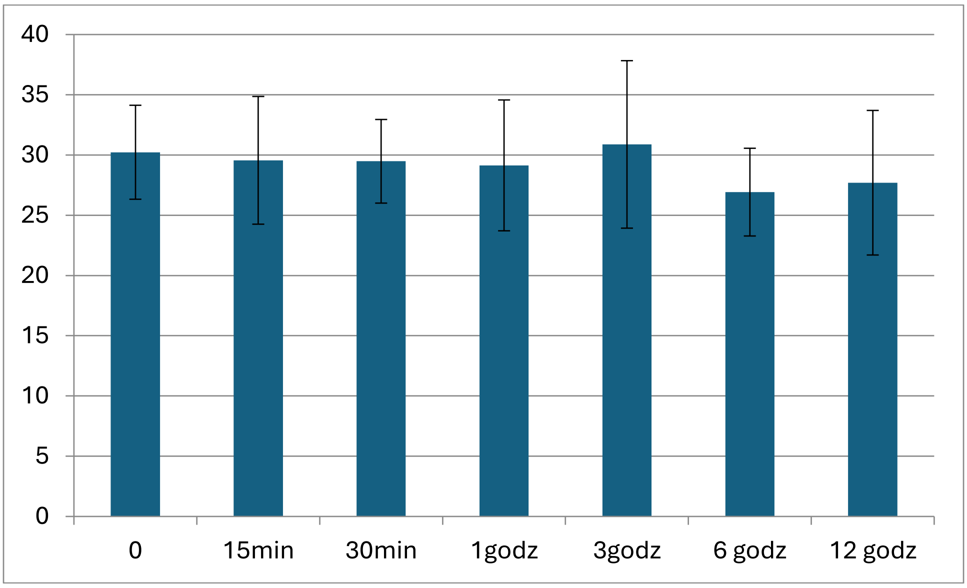

Fig. S3. Graph of the thickness of reproductive layer of epidermis with standard deviation for MTC-U

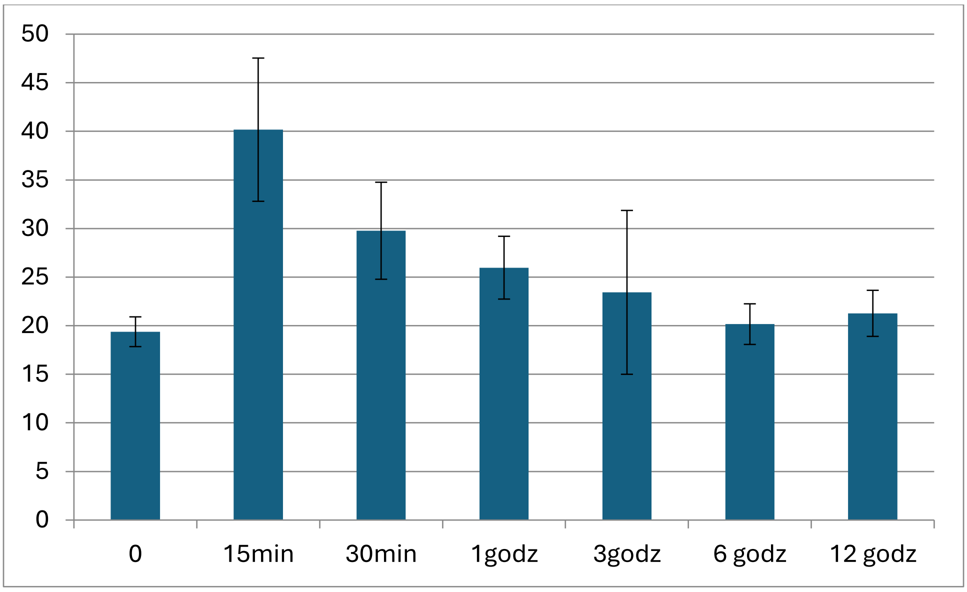

Fig.S4. Graph of the thickness of the keratinized layer of epidermis with standard deviation for MTC-U(Na)

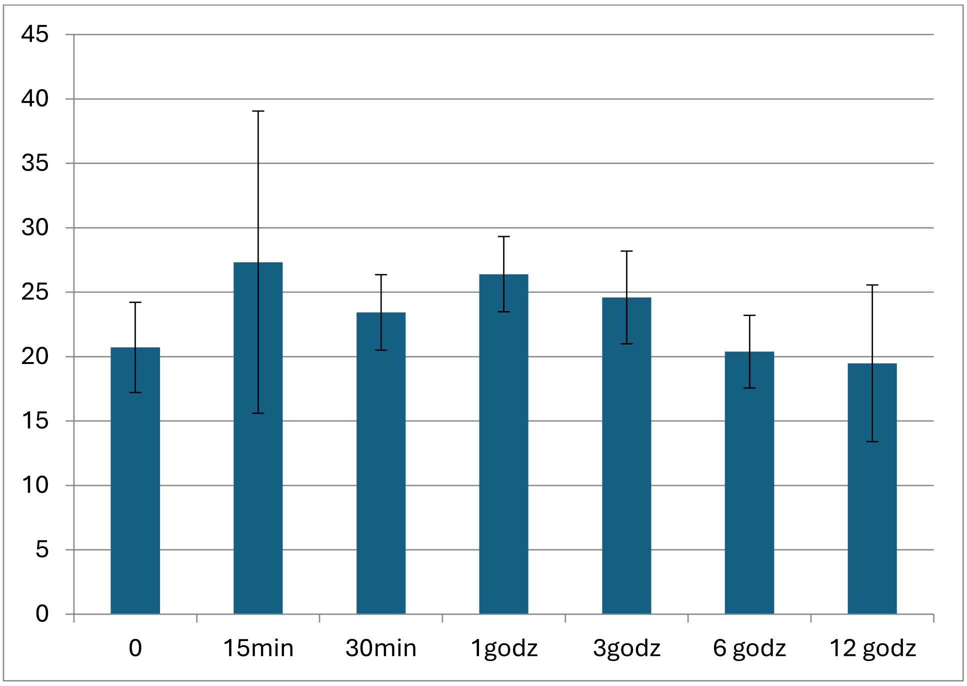

Fig. S5. Graph of the thickness of reproductive layer of epidermis with standard deviation for MTC-U(Na)

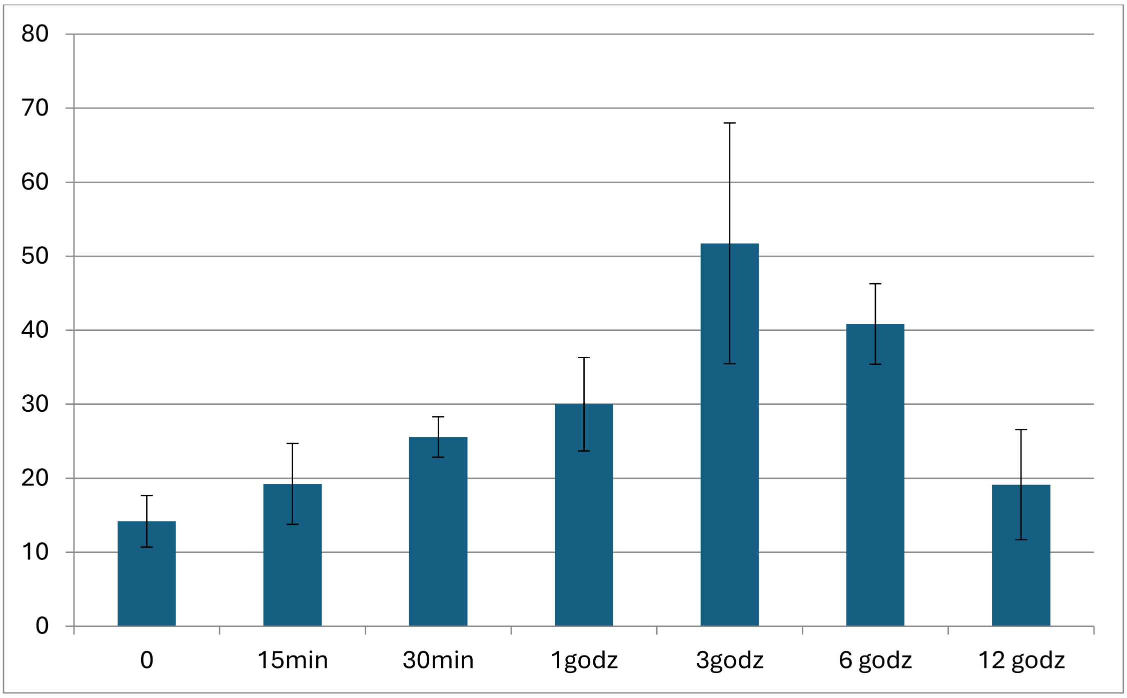

Fig. S6. Graph of the thickness of the keratinized layer of epidermis with standard deviation for MTC-U(A)

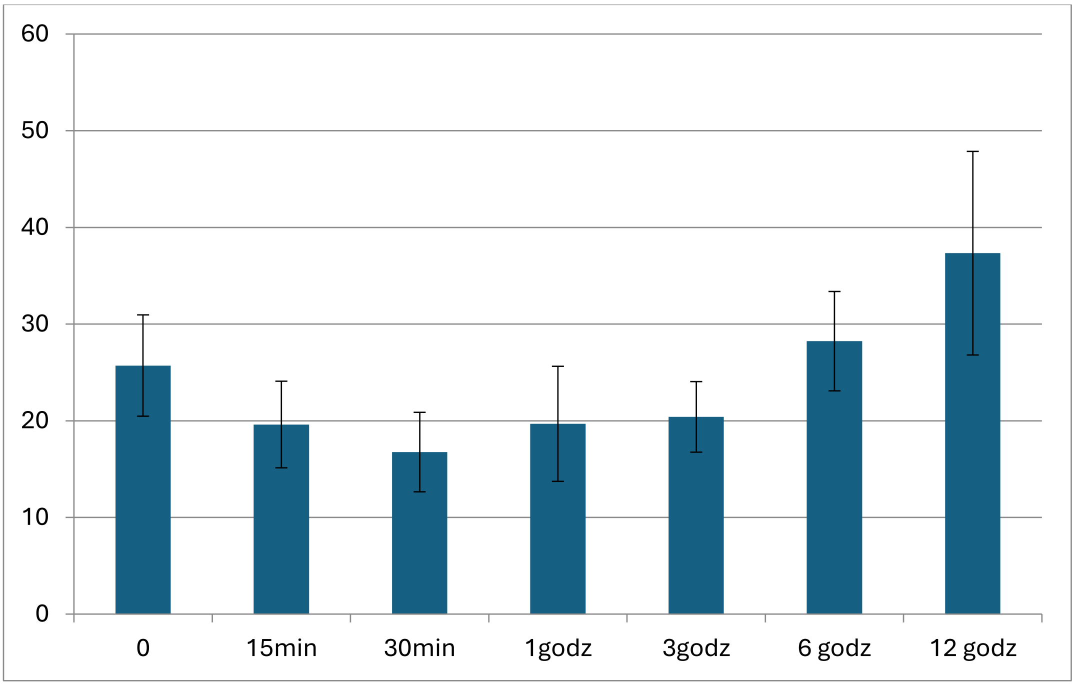

Fig. S7. Graph of the thickness of reproductive layer of epidermis with standard deviation deviation for MTC-U(A)

In vivo analysis

Table. S2. Wound healing based on the scoring in individual groups evaluated from the day of separation of necrotic tissue

Time (days)	Oxytetracycline grup		MTC-U1	
	Mean	SD	Mean	SD
1	15,29	0,49	12,29	3,04
2	15,14	0,69	9,29	3,35
3	13,86	2,41	6,57	1,40
4	15,00	3,42	6,17	2,04
5	12,57	2,07	4,57	2,30
6	9,8	2,27	4,00	2,38
7	9,00	1,00	2,86	0,90
8	7,43	1,13	3,00	2,83
9	5,43	1,27	2,57	1,51
10	3,86	1,46	2,43	1,81
11	3,14	0,38	2,00	1,83
12	3,57	0,98	1,86a	1,46
13	3,00	1,00	1,57	1,13
14	1,86	0,90	1,29	0,49

Table S3. Mean wound healing time counted from the day of separation of necrotic tissue to the formation of scab (assessment of the size of 1/5th of the wound), without the occurrence of swelling, redness, purulent leakage and serous exudate.

Oxytetracycline group		MTC-U1	
Mean	SD	Mean	SD
13,43	2,88	9,57	2,64

Table S4. Thermographic evaluation: Mean temperature of ROI of the tail base area in individual groups from the day of separation of necrotic tissue

Time (days)	Oxytetracycline group		MTC-U1		p-value
	Mean	SD	Mean	SD	
1	40,07	0,67	38,87	1,44	0,78
2	39,86[a]	0,70	37,21	2,34	0,02
3	38,94	0,91	37,07	2,79	0,04

Table S5. Thermographic evaluation: maximum ROI temperature of the tail base area in individual groups on the first 3 days after separation of necrotic tissue

Time (days)	Oxytetracycline group		MTC-U1		p-value
	Mean	SD	Mean	SD	
1	40,64	0,50	39,57	1,34	0,19
2	40,60	0,50	38,43	2,62	0,09
3	39,87	0,63	37,84	2,97	0,07

Table S6. Thermographic evaluation of wound healing: mean temperature of tail tip ROI in individual groups on the first 3 days after separation of necrotic tissue

Time (days)	Oxytetracycline group		MTC-U1		p-value
	Mean	SD	Mean	SD	
1	35,53	3,64	35,71	1,37	0,91
2	34,50	1,85	35,24	2,48	0,41
3	35,54	1,67	34,83	3,45	0,46

Table S7. Thermographic evaluation of wound healing: minimum tail tip ROI temperature in individual groups on the first 3 days after separation of necrotic tissue

Time (days)	Oxytetracycline group		MTC-U1		p-value
	Mean	SD	Mean	SD	
1	31,96	5,92	34,64	1,44	0,47
2	30,14	6,45	33,57	2,90	0,17
3	34,51	1,68	33,70	3,56	0,62

Table S8. Macroscopic evaluation of wound healing progression from day 1 to day 21

Day 1			
MTC-U1	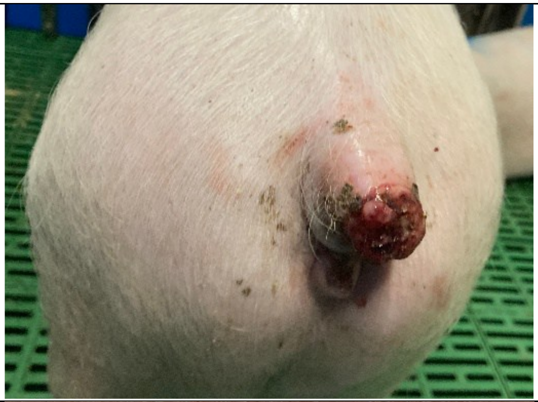	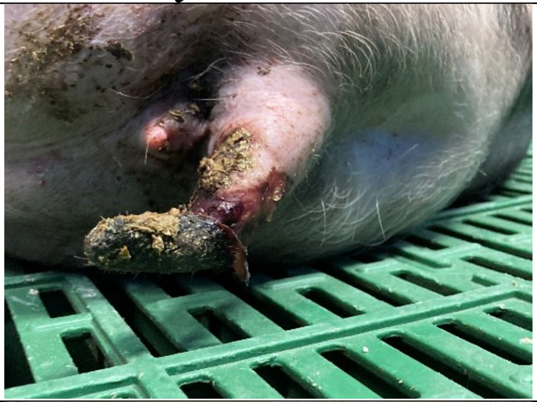	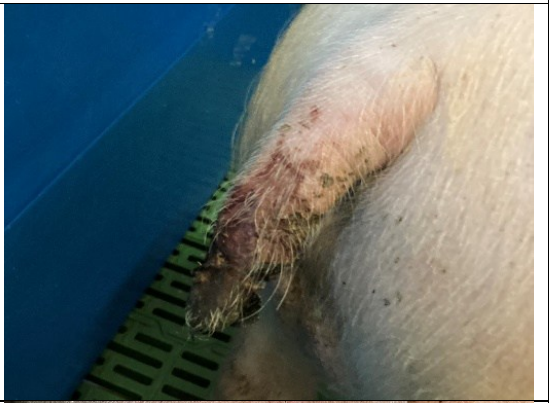
Oxytetracycline spray	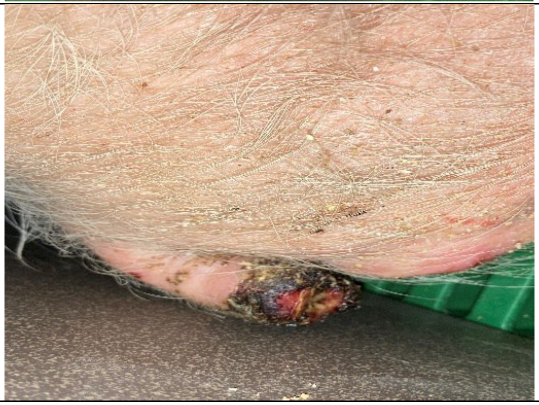	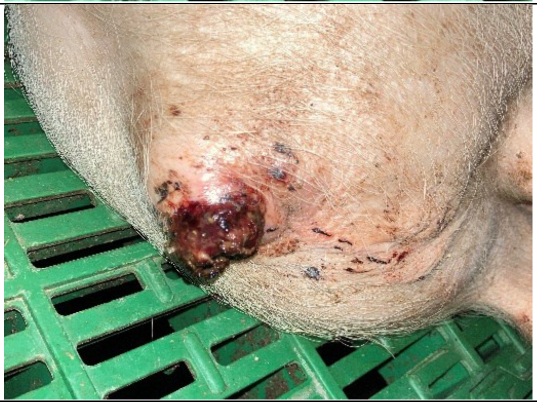	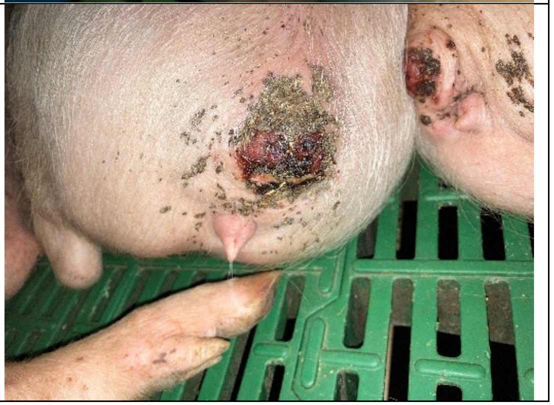

Day 2			
MTC-U1	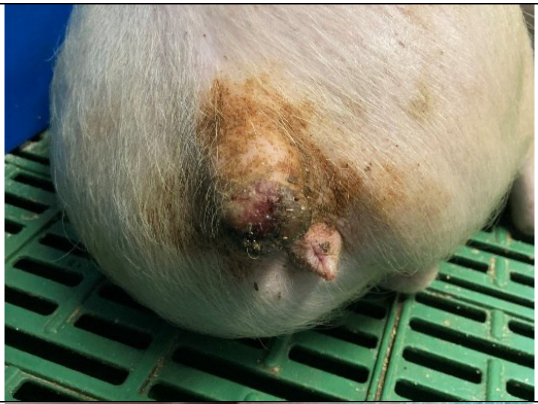	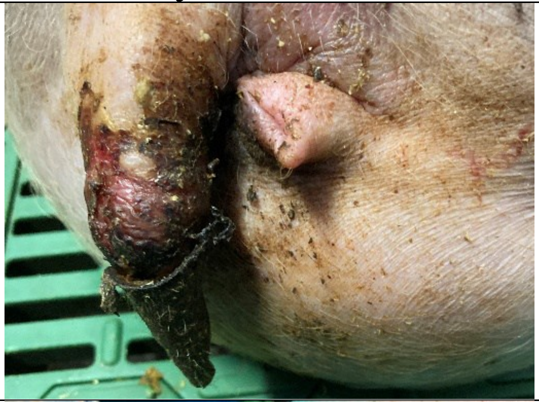	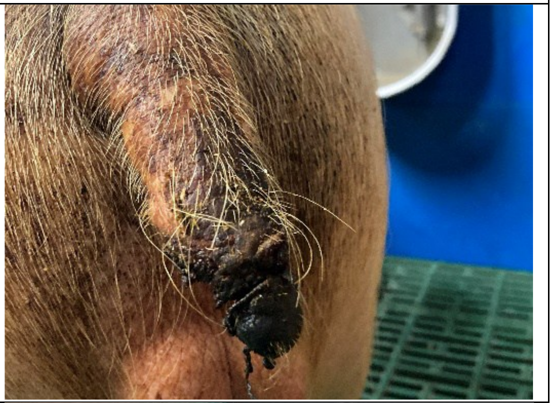
Oxytetracycline spray	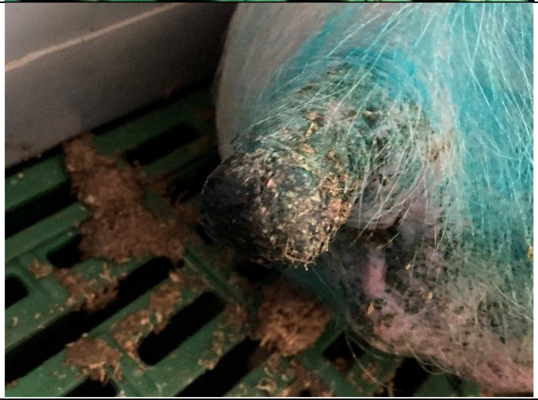	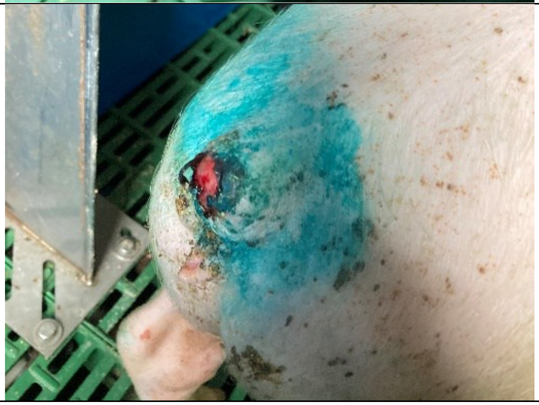	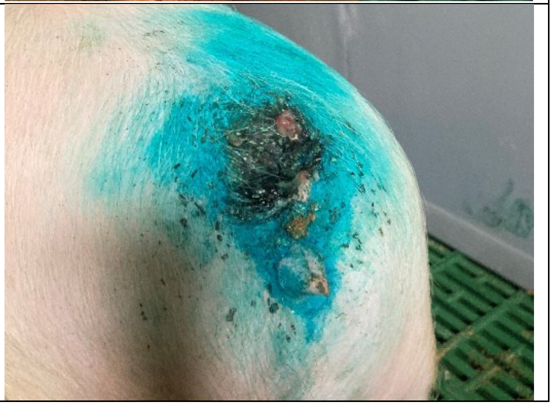

Day 3			
MTC-U1	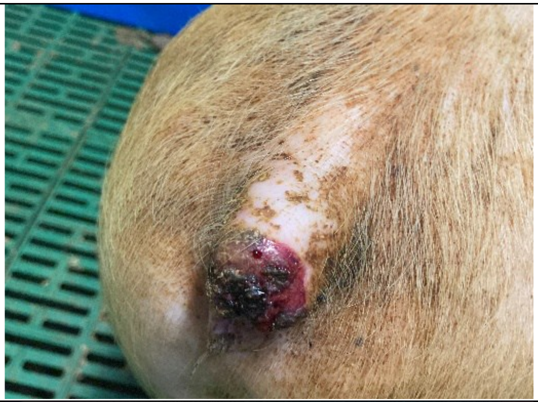	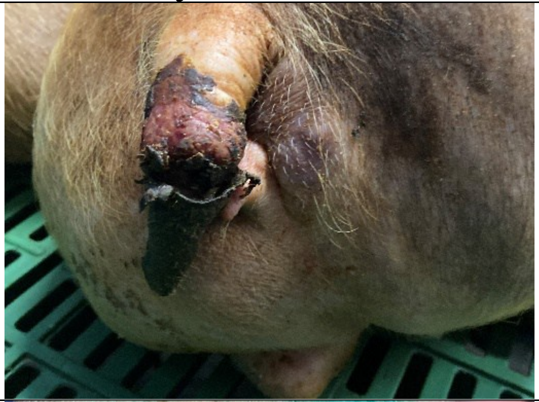	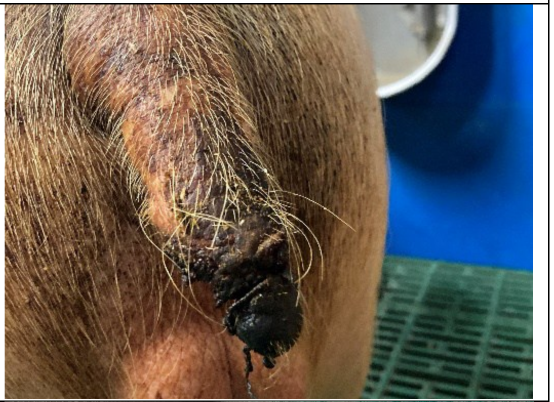
Oxytetracycline spray	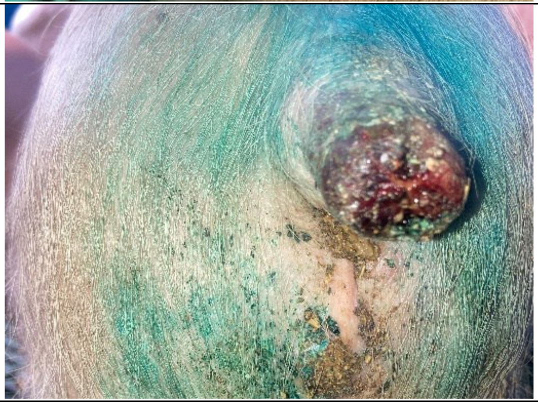	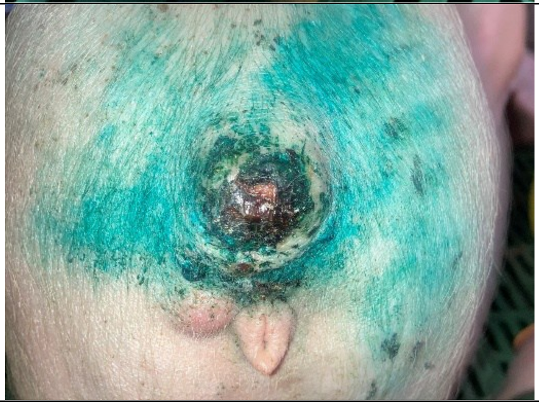	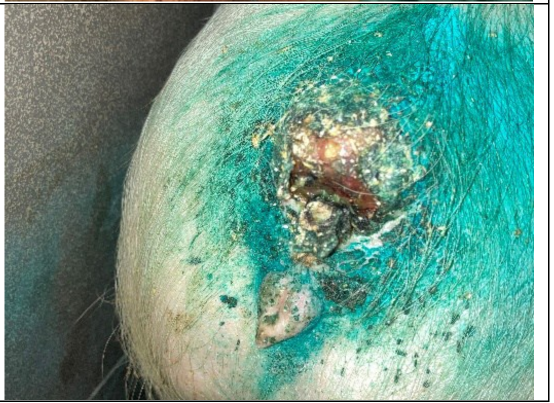

Day 4			
MTC-U1	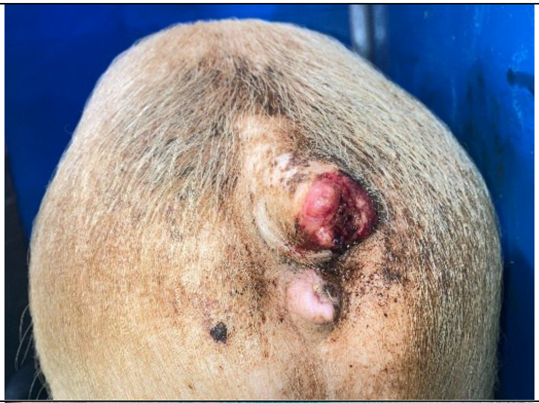	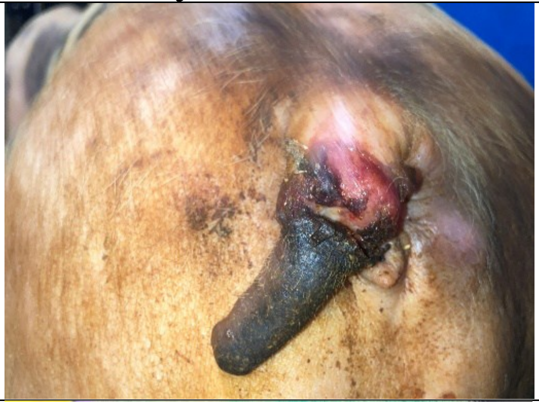	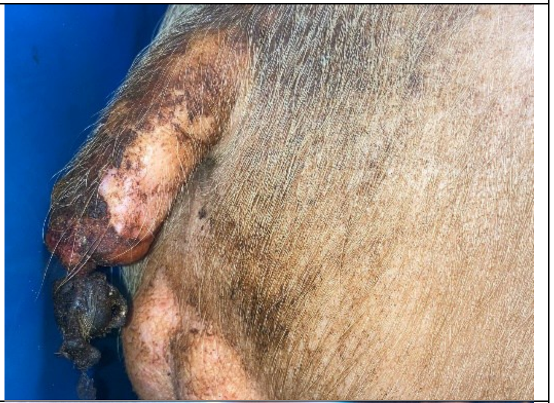
Oxytetracycline spray	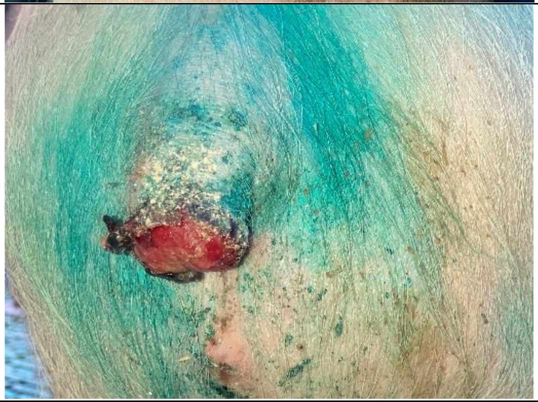	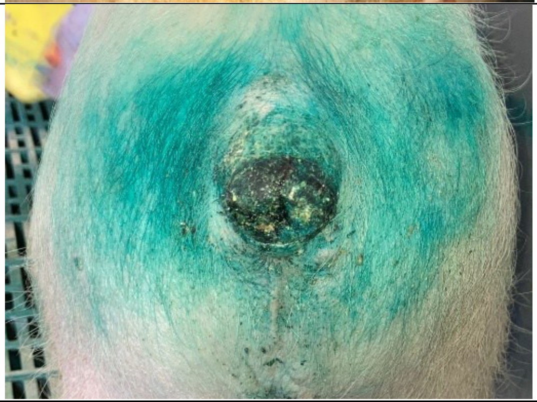	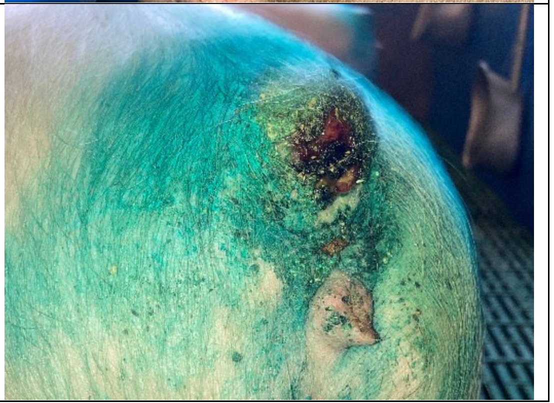

Day 5			
MTC-U1	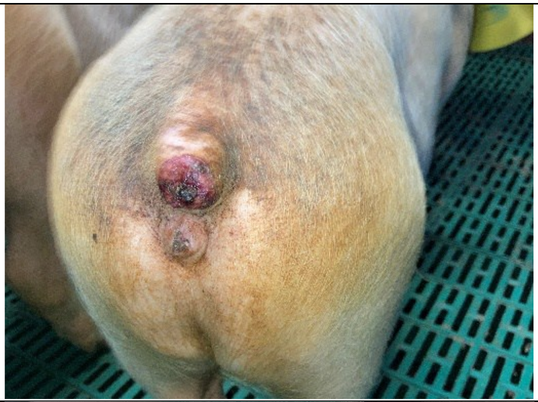	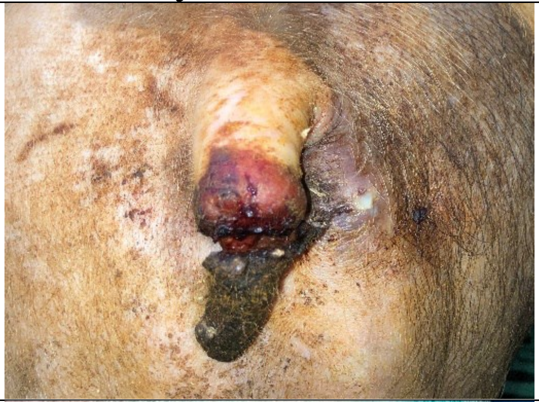	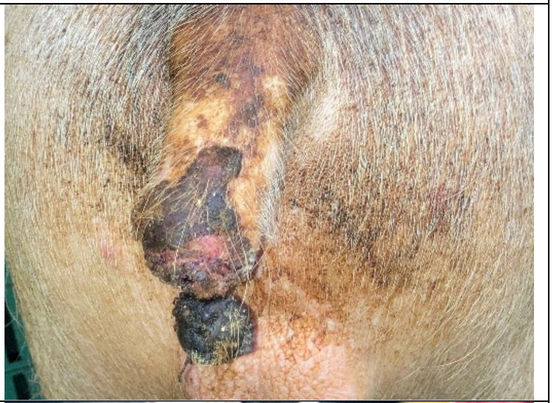
Oxytetracycline spray	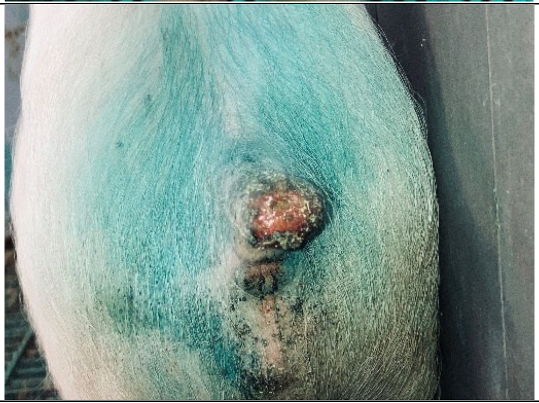	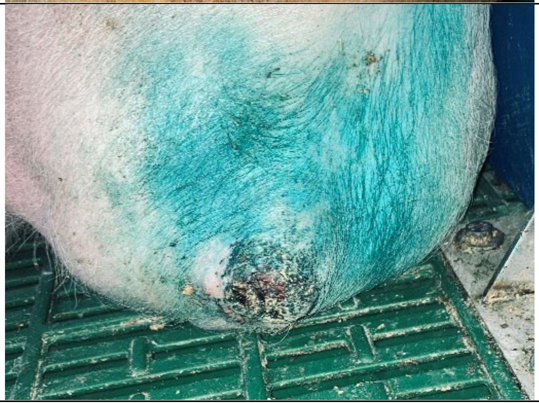	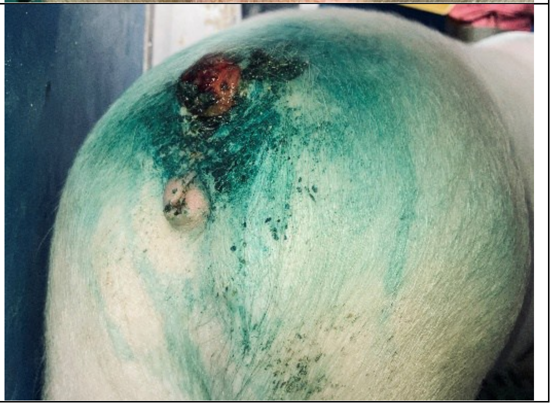

Day 6			
MTC-U1	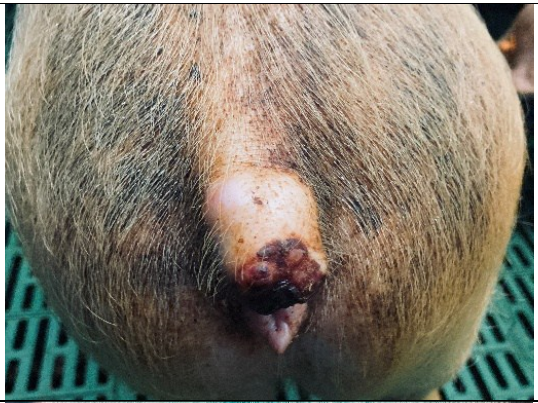	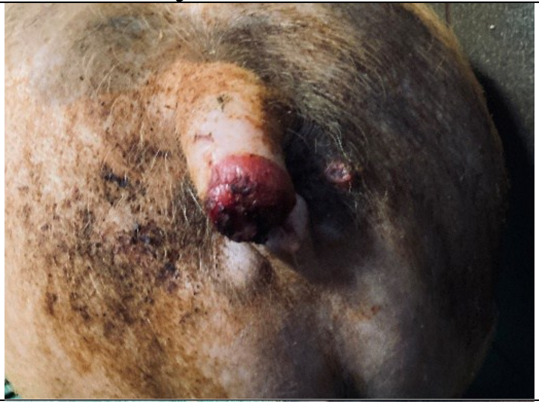	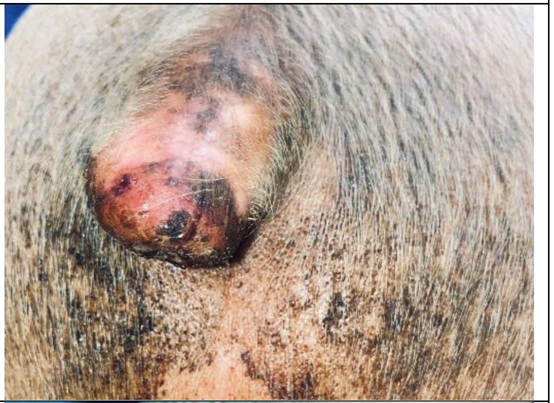
Oxytetracycline spray	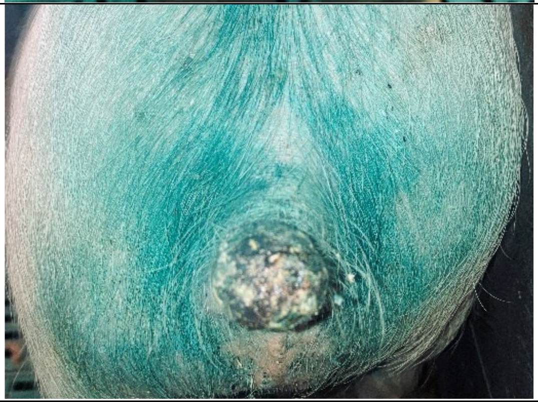	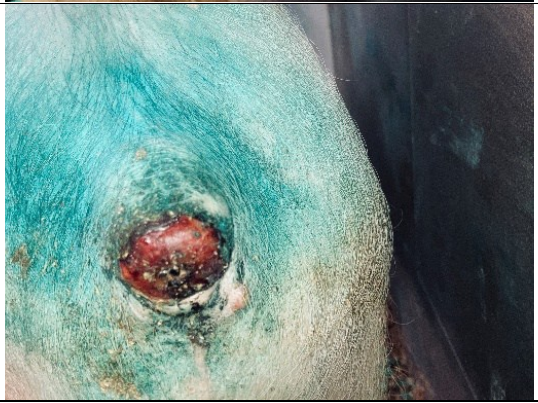	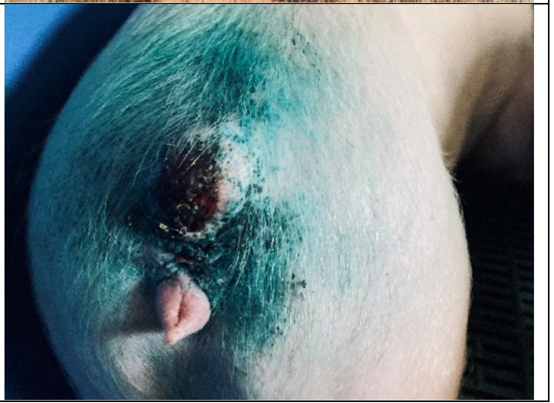

Day 7			
MTC-U1	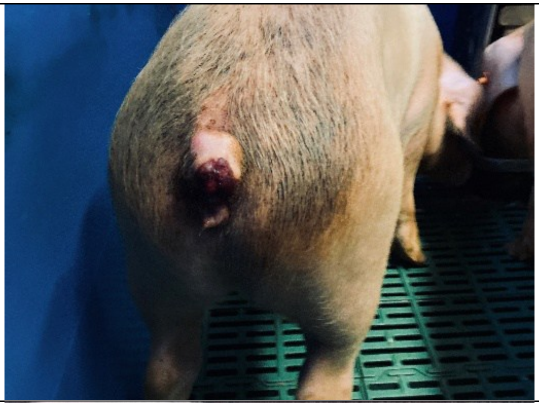	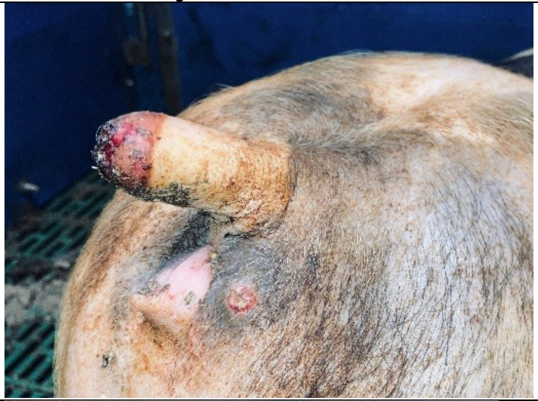	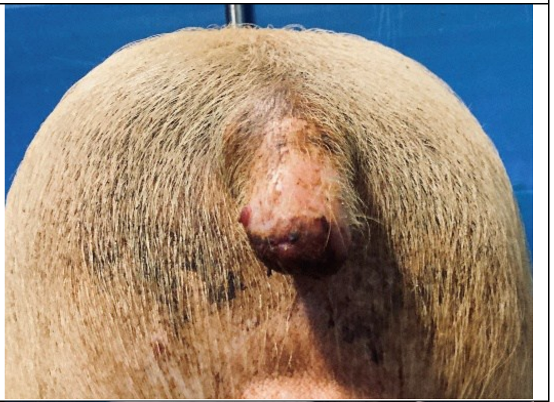
Oxytetracycline spray	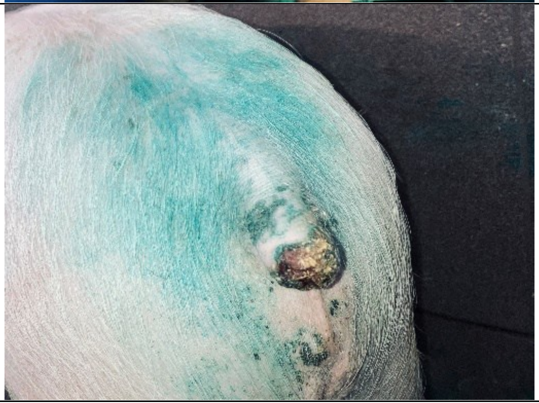	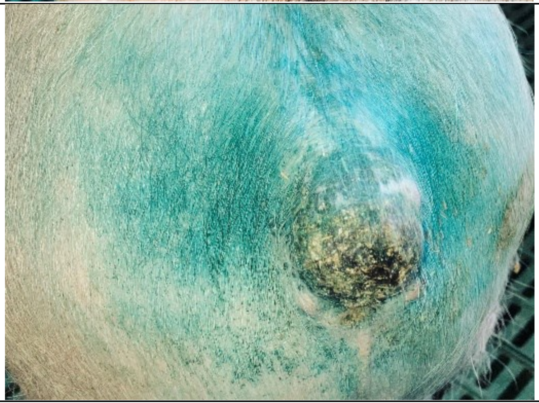	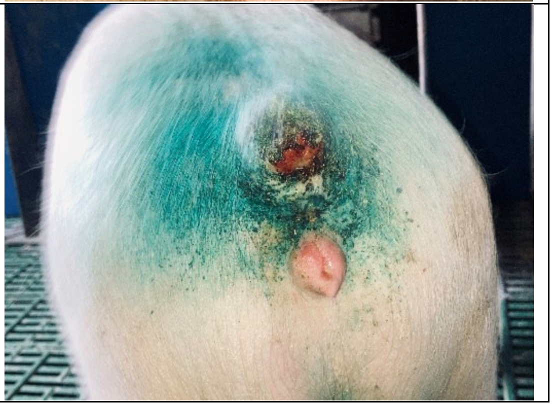

Day 9			
MTC-U1	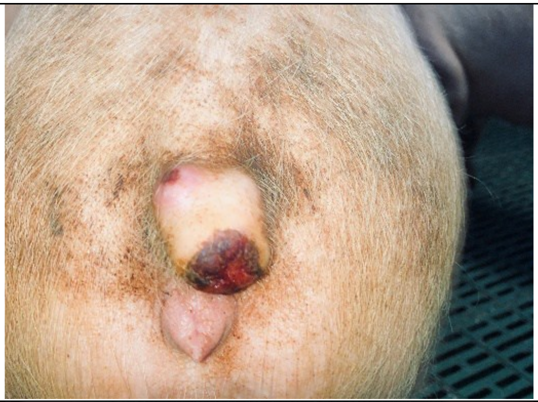	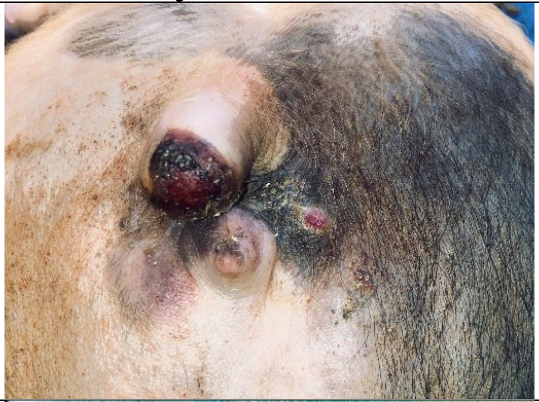	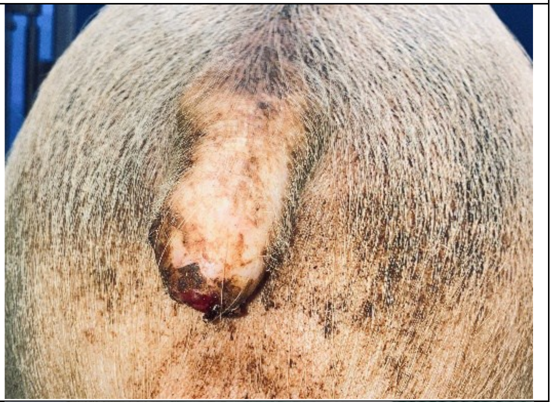
Oxytetracycline spray	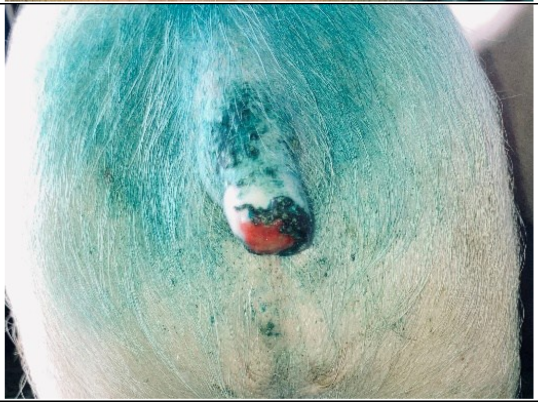	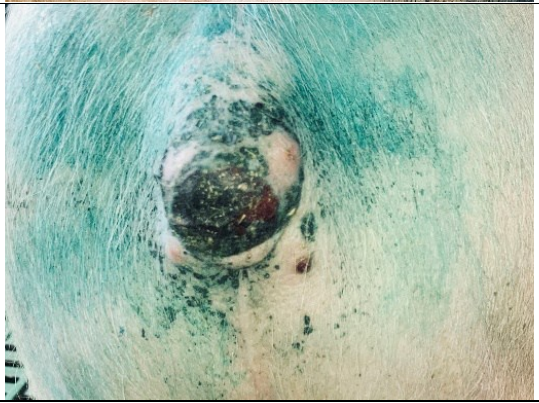	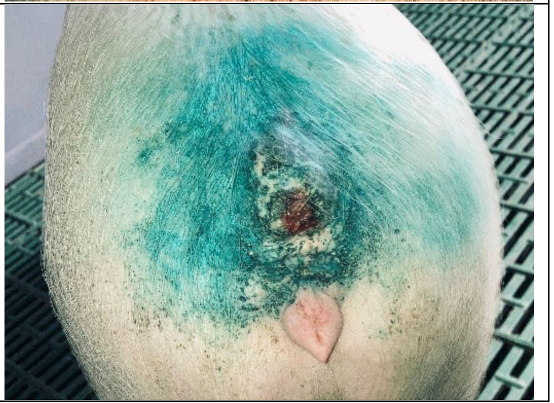

Day 11			
MTC-U1	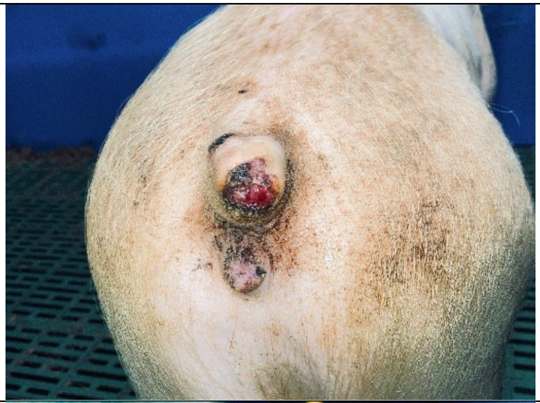	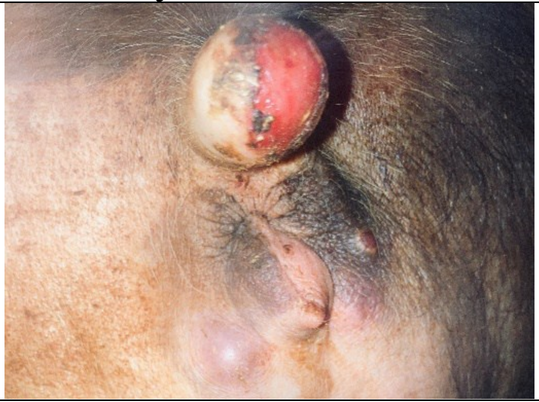	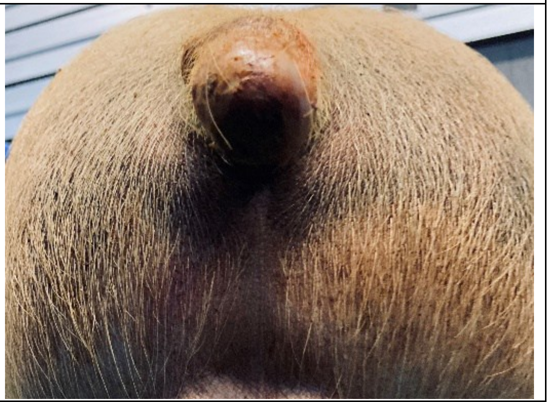
Oxytetracycline spray	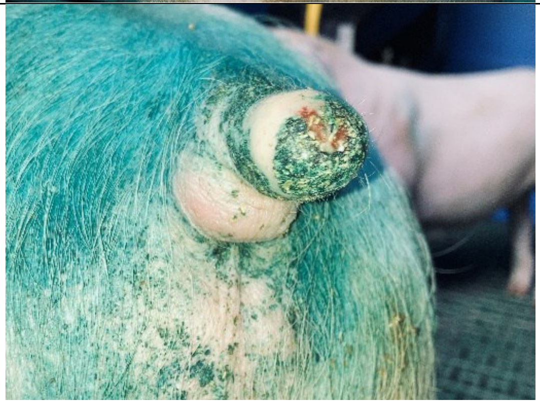	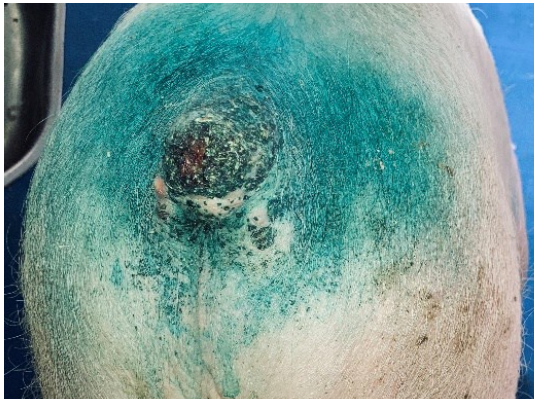	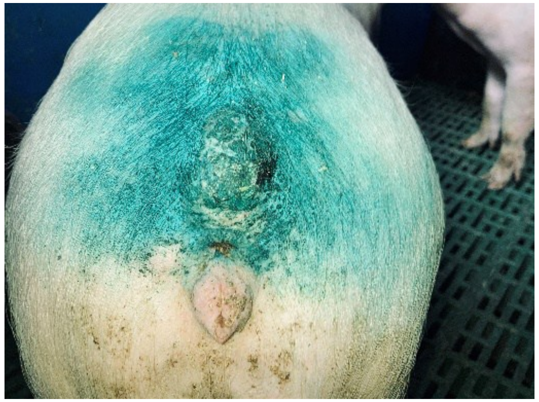

Day 13			
MTC-U1	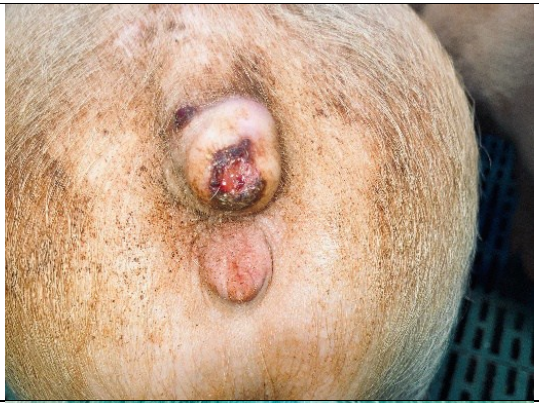	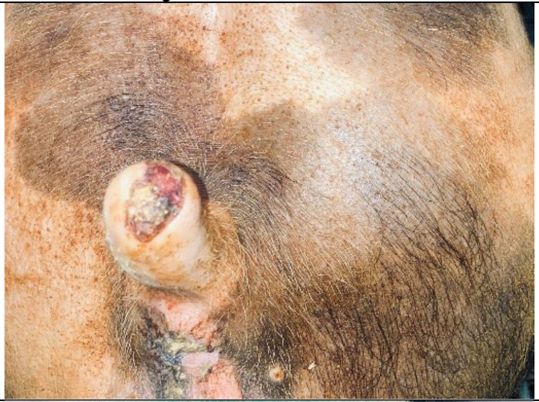	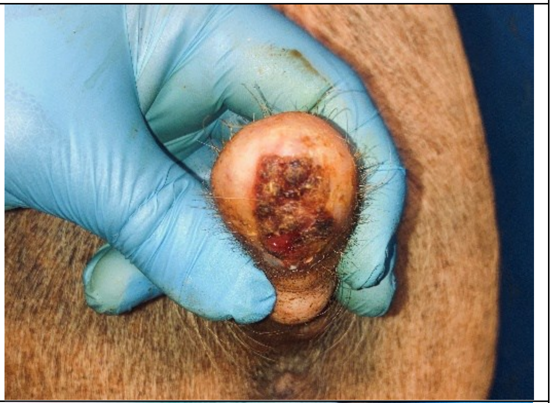
Oxytetracycline spray	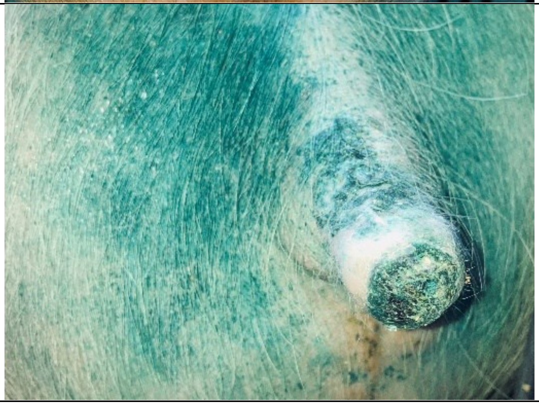	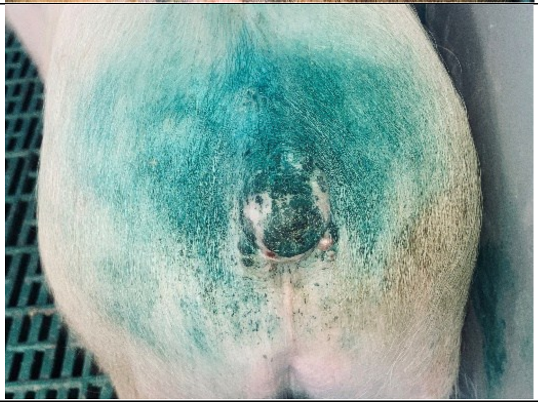	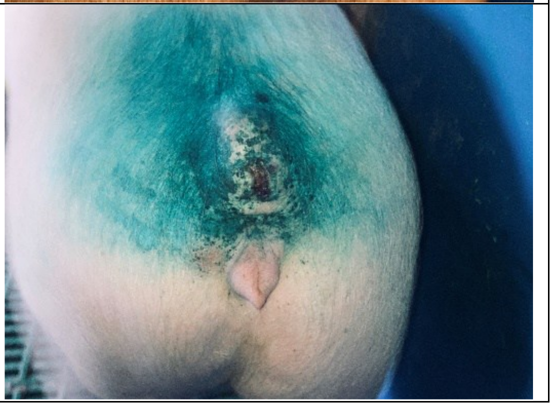

Day 15			
MTC-U1	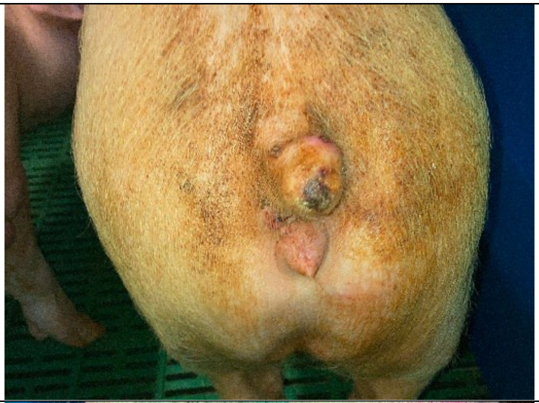	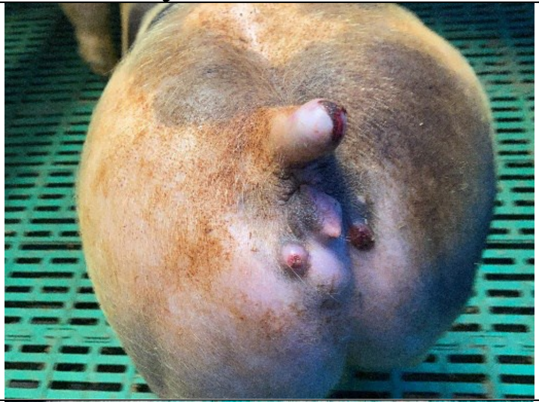	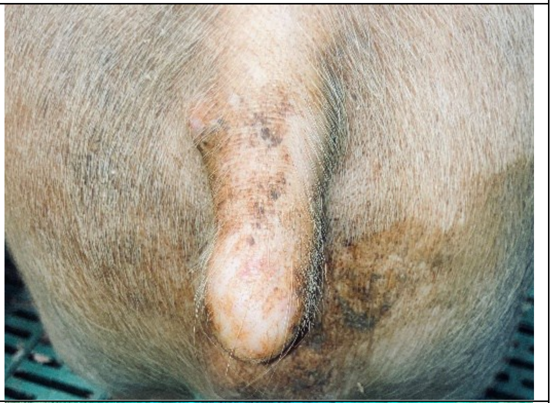
Oxytetracycline spray	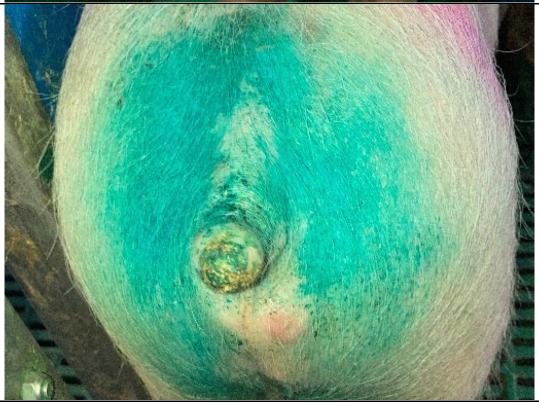	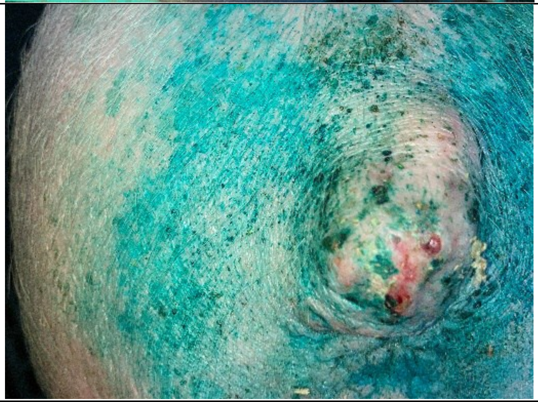	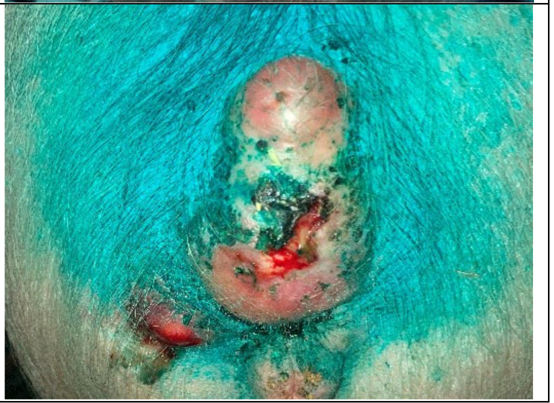

Day 17			
MTC-U1	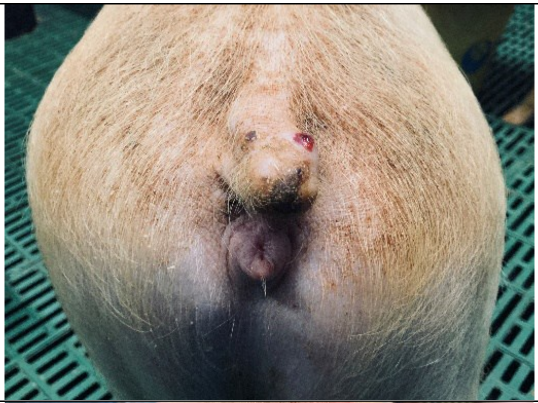	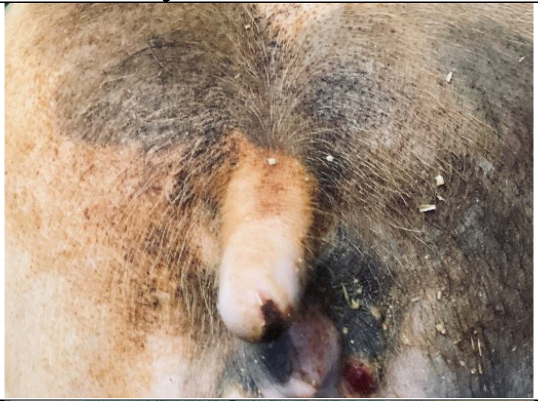	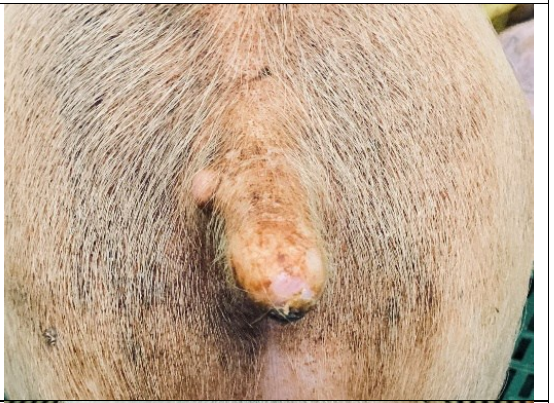
Oxytetracycline spray	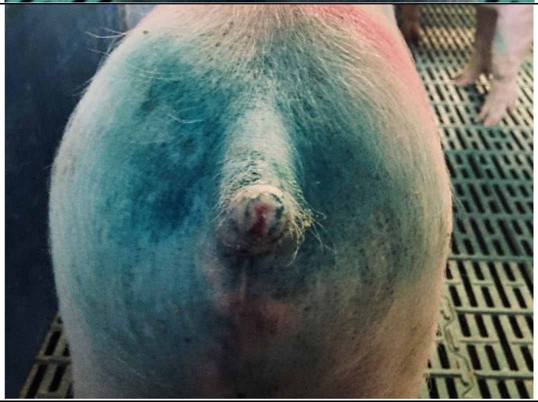	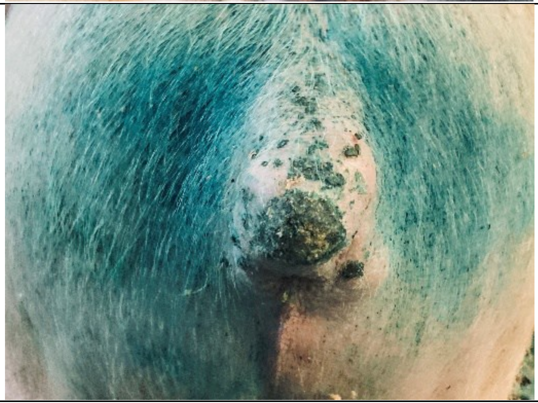	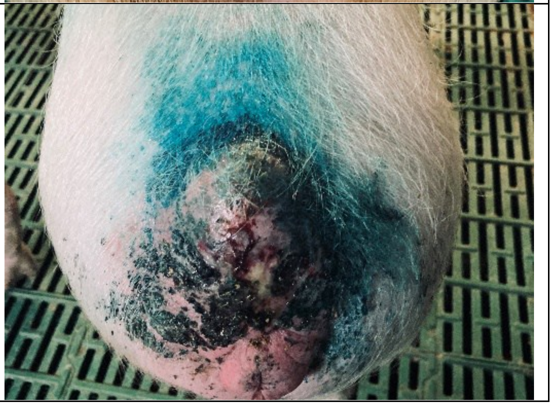

Day 19			
MTC-U1	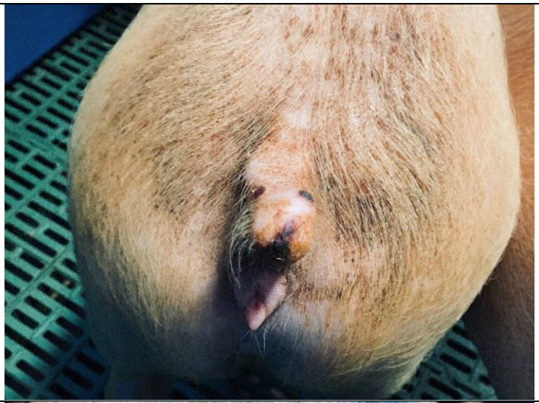	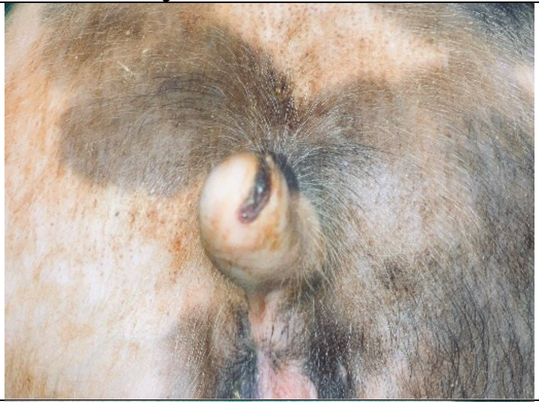	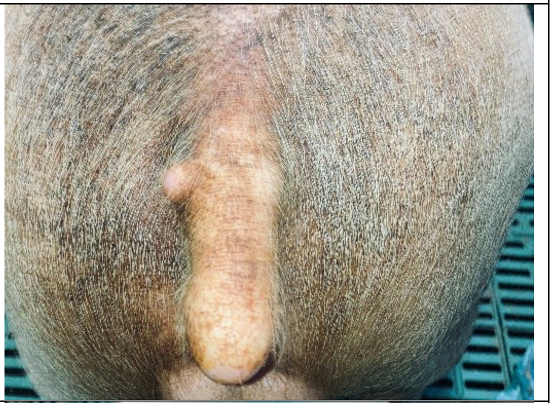
Oxytetracycline spray	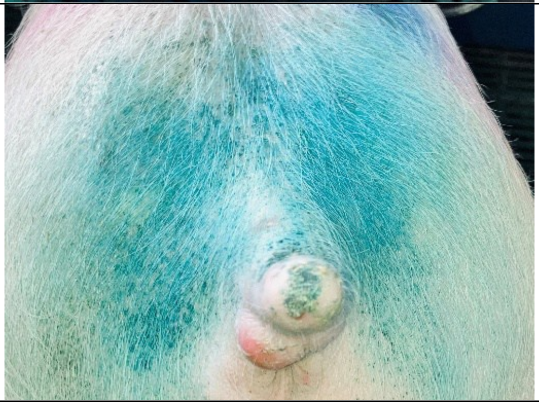	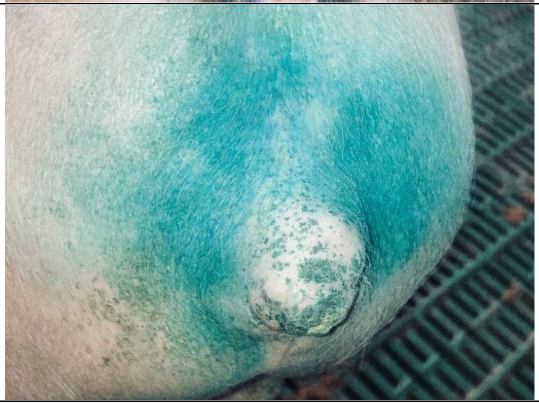	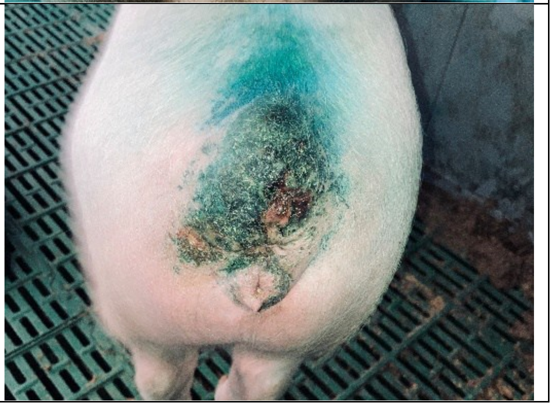

Day 21			
MTC-U1	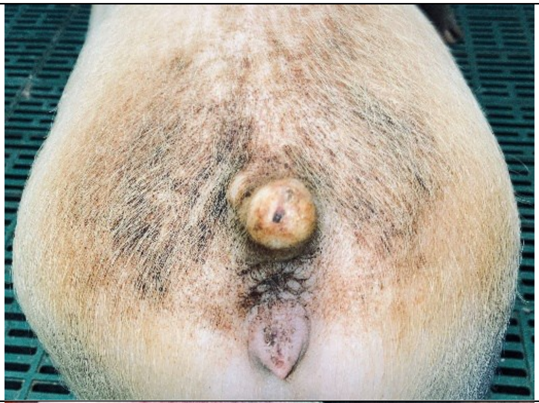	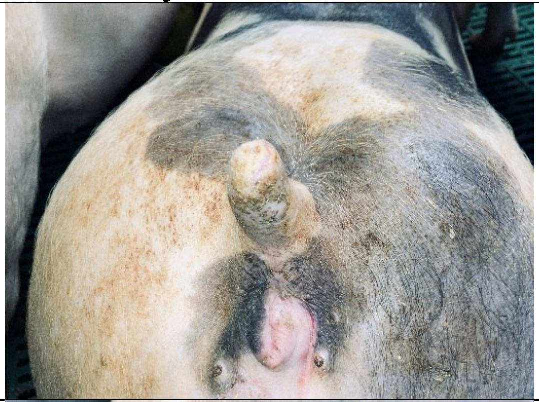	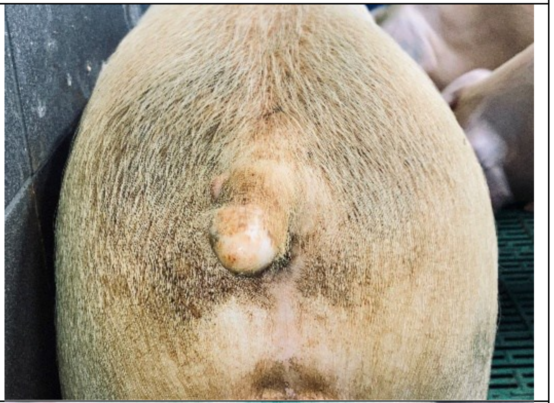
Oxytetracycline spray	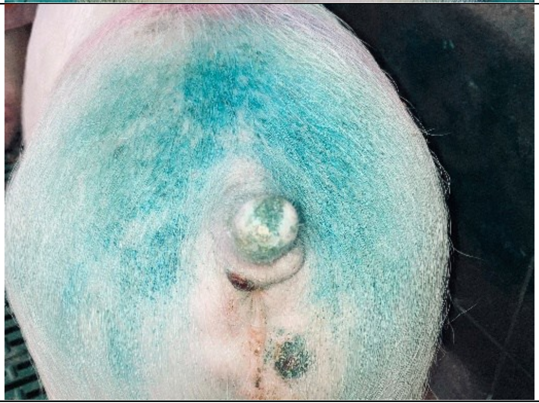	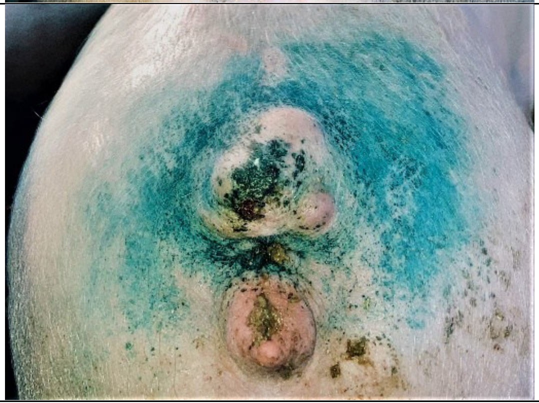	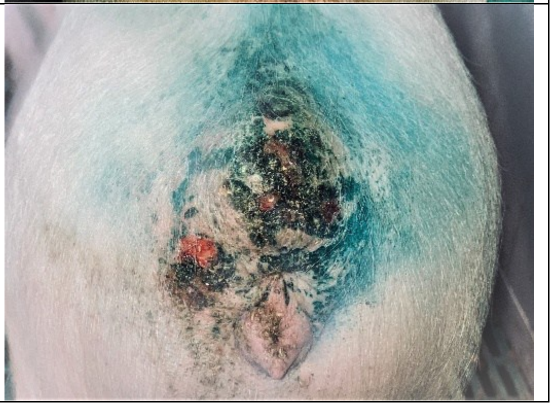

Table S9. Mean number of leukocytes in examined animals in particular groups depending on the time of intake.

Value	Intake	Oxytetracycline group		MTC-U1		Standard range
		Mean	SD	Mean	SD	
Absolute (x 10^9/L)	I	27,98AC	1,27	28,48B	3,48	11,0-22,0

Table S10. Relative and absolute number of neutrophils in tested animals in individual groups depending on the time of intake.

Value	Intake	Oxytetracycline group		MTC-U1		Standard range
		Mean	SD	Mean	SD	
Absolute (x 10^9/L)	I	12,18	3,88	14,54a	1,26	3,0-10,0
	II	12,70	4,97	8,79a	2,48	
Relative	I	45,53%	11,13%	53,73%	0,60%	28–47%
	II	47,95%	9,82%	37,11%	7,05%	

Table S11. Relative and absolute number of lymphocytes in examined animals in particular groups depending on the time of intake.

Value	Intake	Oxytetracycline group		MTC-U1		Standard range
		Mean	SD	Mean	SD	
Absolute (x 10^9/L)	I	12,49ab	2,72	8,96aA	2,36	4,2-13,6
	II	10,15	1,00	12,36A	1,03	
Relative	I	42,43%	9,76%	29,53%	2,71%	39–62 %
	II	45,97%	9,72%	53,48%	7,86%	

Table S12. Relative and absolute number of monocytes in experimental groups according to the sampling time.

Value	Intake	Oxytetracycline group		MTC-U1		Standard range
		Mean	SD	Mean	SD	
Absolute (x 10^9/L)	I	2,28B	0,21	2,89AC	1,02	0,2-2,2
	II	0,59B	0,09	0,97C	0,22	
Relative	I	8,21%	0,66%	9,33%	1,85%	2–10
	II	2,72%	0,47%	3,99%	0,84%	

Table S13. Relative and absolute number of acid-absorbent granulocytes (eosinophils) in experimental groups according to the sampling time.

Value	Intake	Oxytetracycline group		MTC-U1		Standard range
		Mean	SD	Mean	SD	
Absolute (x 10^9/L)	I	0,52Ac	0,08	1,66AB	0,51	0,5-2,4
	II	0,61c	0,19	0,98	0,36	
Relative	I	1,80%	0,24%	5,92%	1,49%	0.5–11
	II	2,55%	0,41%	4,45%	0,83%	

Table S14. Relative and absolute number of alkaline granulocytes (basophils) in tested animals in particular groups depending on the time of collection.

Value	Intake	Oxytetracycline group		MTC-U1		Standard range
		Mean	SD	Mean	SD	
Absolute (x 10^9/L)	I	0,28AB	0,09	0,41A	0,07	0-0,4
	II	0,19	0,10	0,22	0,06	
Relative	I	0,96%	0,29%	1,48%	0,28%	0–2
	II	0,81%	0,31%	0,88%	0,26%	

Table S15. Protein parameters as indicators of inflammation in examined animals in particular groups.

Parameter	Oxytetracycline group		MTC-U1		Standard range
	Mean	SD	Mean	SD	
Total protein (g/L)	64,23	3,76	64,66	5,01	35-60
Albumines (g/L)	23,61	2,96	20,36	2,52	19-24
Globulines (g/L)	40,61	4,68	44,30	6,76	-
Albumin/globulin ratio	0,59	0,12	0,48	0,13	0,59-1,13

Table S16. Mean concentrations of acute phase proteins (haptoglobin and fibrinogen) in the examined groups of animals.

Parameter	Oxytetracycline group		MTC-U1		Standard range
	Mean	SD	Mean	SD	
Haptoglobin (mg/ml)	1,86a	0,87	0,74a	0,66	~ 1
Fibrinogen (g/L)	4,04	0,25	4,76	0,92	1-5

Table S17. Weight production indicators

Value	Oxytetracycline group		MTC-U1	
	Mean	SD	Mean	SD
Initial weight (kg)	25,32	2,16	31,46	6,26
Final weight (kg)	34,91	4,58	45,71	6,01
Increase (kg within 21 days)	9,59	5,51	14,26	0,64

Table S18. Basic production indicators: daily increments, mean daily feed intake and feed conversion rate.

	Oxytetracycline group	MTC-U1
ADG (Mean daily gain), g/d	456,67	678,91
Mean daily feed consumption	775,86[a]	976,91
FCR (feed conversion ratio)	1,69	1,44
